# Harnessing copper-palladium alloy tetrapod nanoparticle-induced pro-survival autophagy for optimized photothermal therapy of drug-resistant cancer

**DOI:** 10.1038/s41467-018-06529-y

**Published:** 2018-10-12

**Authors:** Yunjiao Zhang, Rui Sha, Lan Zhang, Wenbin Zhang, Peipei Jin, Weiguo Xu, Jianxun Ding, Jun Lin, Jing Qian, Guangyu Yao, Rui Zhang, Fanchen Luo, Jie Zeng, Jie Cao, Long-ping Wen

**Affiliations:** 1 Department of Colorectal & Anal Surgery, Guangzhou First People’s Hospital, School of Medicine, South China University of Technology, Guangzhou, 510006 Guangdong China; 2 Nanobio Laboratory, Institute of Life Sciences, South China University of Technology, Guangzhou, 510006 Guangdong China; 3 School of Life Sciences, University of Science and Technology of China, Hefei, Anhui 230027 P. R. China; 4 Hefei National Laboratory for Physical Sciences at the Microscale, Center of Advanced Nanocatalysis (CAN-USTC) and Department of Chemical Physics, University of Science and Technology of China, Hefei, Anhui 230026 P. R. China; 5 Key Laboratory of Polymer Ecomaterials, Changchun Institure of Applied Chemistry, Chinese Academy of Sciences, Jilin, 130022 Changchun P. R. China; 6 Breast Center, Nanfang Hospital, Southern Medical University, Guangzhou, 510515 Guangdong China

## Abstract

Chemo-PTT, which combines chemotherapy with photothermal therapy, offers a viable approach for the complete tumor eradication but would likely fail in drug-resistant situations if conventional chemotherapeutic agents are used. Here we show that a type of copper (Cu)-palladium (Pd) alloy tetrapod nanoparticles (TNP-1) presents an ideal solution to the chemo-PTT challenges. TNP-1 exhibit superior near-infrared photothermal conversion efficiency, thanks to their special sharp-tip structure, and induce pro-survival autophagy in a shape- and composition-dependent manner. Inhibition of autophagy with 3-methyl adenine or chloroquine has a remarkable synergistic effect on TNP-1-mediated PTT in triple-negative (4T1), drug-resistant (MCF7/MDR) and patient-derived breast cancer models, achieving a level of efficacy unattainable with TNP-2, the identically-shaped CuPd nanoparticles that have a higher photothermal conversion efficiency but no autophagy-inducing activity. Our results provide a proof-of-concept for a chemo-PTT strategy, which utilizes autophagy inhibitors instead of traditional chemotherapeutic agents and is particularly useful for eradicating drug-resistant cancer.

## Abstract (web summary)

“Conventional chemotherapy-photothermal therapy combination has limited efficacy in drug resistant cancers. Here they develop Copper-palladium tetrapod nanoparticles to overcome these challenges and show them to work in synergy with autophagy inhibitors to treat drug resistant cancers”

## Introduction

A major cause of failure in the treatment of cancer is the presence or development of drug resistance by the cancer cells^1,2^. This is one of the most pressing problems affecting the majority of cancer patients, leading to recurrence of disease and eventually death of patients from distant metastases that have become resistant to all available chemotherapy. Current treatment options are highly inadequate in addressing this problem, and novel approaches for overcoming cancer drug resistance are urgently needed. A promising approach, among many that are actively pursued, is photothermal therapy (PTT). In this noninvasive approach, tumor cells are killed by thermal energy generated from photothermal agents after adsorbing light^3–5^, thus offering an alternative mechanism to elicit cancer cell death. A variety of nanoparticles, such as gold nanostructures^6,7^, graphene^8,9^ and other carbon nanomaterials^10^, palladium (Pd) nanosheets^11,12^, copper (Cu) based nanoparticles^13,14^, metal-organic frameworks^15^, and polymer nanomicelles encapsulated with near-infrared (NIR) dyes^16^, have been extensively investigated as NIR^−^assisted photothermal agents, and some of them have demonstrated outstanding results. However, a common issue with nanomaterial-mediated PTT is the inability to induce complete tumor eradication due to the non-uniform distribution of hyperthermia^17–19^. Combining PTT with chemotherapy, termed chemo-photothermal therapy (chemo-PTT), provides a feasible strategy for resolving this issue, thanks to the complementary, and possibly synergistic, cancer-killing effects elicited by the two approaches ^20,21^. However, the majority of the chemo-PTT studies reported in the literature employ chemotherapeutic agents that are widely used in clinics today, and in these circumstances chemo-PTT would be self-defeating for drug-resistant cancer. Complementing PTT with novel chemotherapeutics, which enhance cancer cell killing through non-conventional mechanisms, would be invaluable for countering the prevalent chemo-resistance problem.

One of the nanomaterials with tremendous potential in NIR^−^assisted PTT is Cu-Pd alloy nanoparticle. Both Cu and Pd nanomaterials display strong LSPR absorption in the spectral range of NIR and could efficiently transfer the absorbed NIR optical energy into heat. Pd nanostructures were recently reported as attractive photothermal agents^11,22–24^, exhibiting higher photothermal conversion efficiency and better photostability than gold or silver nanostructures. Cu-based nanomaterials have also shown great promise in photothermal thearapy^25–31^. The potential benefit of adding Cu to Pd nanoparticle, in addition to tuning photothermal response and reducing cost, is the ability to induce autophagy, a critical cellular degradation process and a key determinant for cancer therapy. Recent studies have shown that Cu compounds and Cu oxide nanoparticles induce autophagy in cancer cells^32,33^. Elevated autophagy oftentimes has a profound impact on the cell fate, promoting either survival or death. Interestingly, and ironically, autophagy induced by Cu compounds elicit both pro-death and pro-survival effects, as the inhibition of autophagy increased cell death for one Cu compound^32^ but decreased cell death for another compound^33^. Depending on the cell fate outcome, the induced autophagy may be differentially exploited to enhance cancer therapy. If the induced autophagy is pro-death, it can be utilized either to directly kill cancer cells^34,35^ or sensitize drug-resistant cancer cells to chemotherapeutic killing^36,37^. On the other hand, if the induced autophagy is pro-survival^38^, inhibitors of autophagy can be used to enhance cancer cell killing, as we demonstrated for silver nanoparticles^39^.

With the above considerations in mind, we propose that a PTT-ready nanomaterial with the capability of inducing pro-survival autophagy may achieve superior therapeutic effect against drug-resistant cancer through the combination of PTT and an autophagy inhibitor, and we demonstrate the feasibility of this chemo-PTT strategy with specially-designed CuPd alloy nanocrystals.

## Results

### Synthesis, characterizations and photo-thermal response of CuPd nanoparticles

**Fig. 1:**
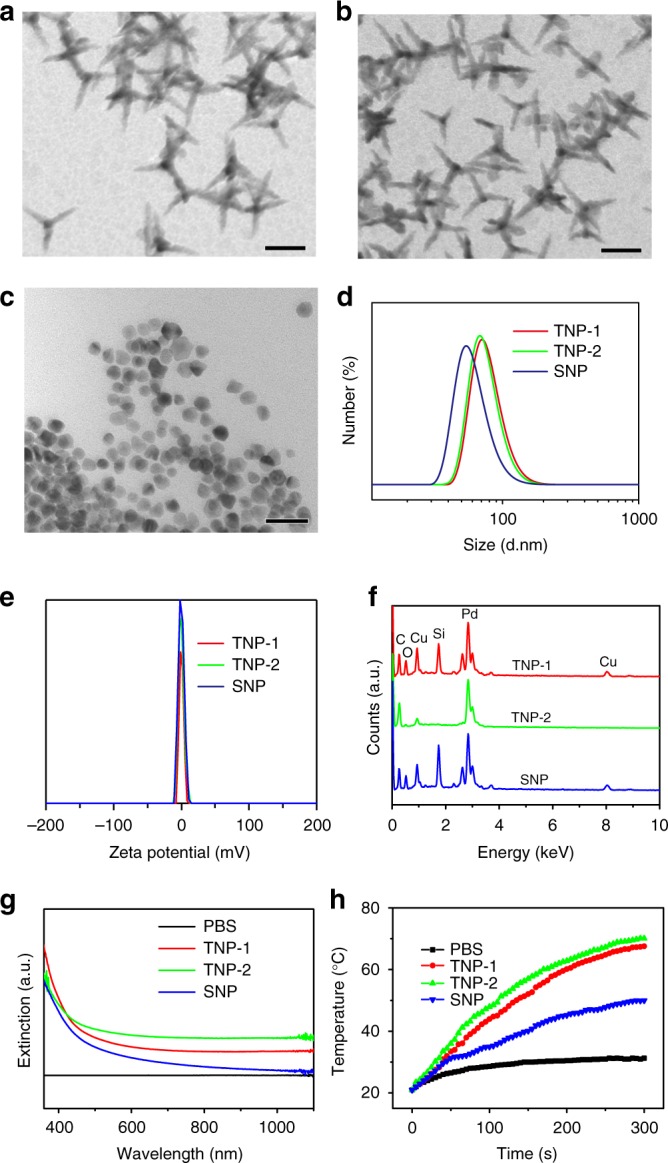
Synthesis and characterizations of CuPd alloy nanoparticles. **a**–**c** TEM images of CuPd TNP-1 (**a**), TNP-2 (**b**) and SNP (**c**). **d** Size distribution of CuPd nanoparticles in aqueous solution as measured by dynamic light scattering (DLS). **e** Zeta potential of CuPd nanopartcles in aqueous solution. **f** Different Cu and Pd contents in CuPd nanoparticles revealed by EDS spectra. **g** The UV-Vis spectrum of CuPd nanoparticles in aqueous solution. **h** The temperature changes of CuPd nanoparticles irradiated by 808 nm NIR laser at a power density of 1 W cm^2^^-1 for 5 min

Three types of CuPd alloy nanoparticles, including two tetrapod nanoparticles (TNP-1 and TNP-2) and one spherical nanoparticle (SNP), were synthesized for the present study. Transmission electron microscopy (TEM) revealed that the two TNPs exhibited very similar size and shape, consisting of four slender arms with ~50 nm in length, while SNP had a slightly smaller size of 35 nm (Fig. 1a, b and c respectively). Dynamic light scattering analysis showed an average size of 68.2, 72.1, and 50.7 nm for TNP-1, TNP-2, and SNP, respectively, in deionized water (Fig. 1d), with the increase likely due to the thickness of the hydration shell. All three CuPd nanoparticles exhibited near-neutral charge in deionized water (Fig. 1e). The EDS spectra (Fig. 1f) showed that the atomic ratio of Cu was 42% for TNP-1, 13% for TNP-2, and 39% for SNP, while the corresponding numbers were 39.7% for TNP-1, 10.3% for TNP-2, and 41.1% for SNP as determined by ICP analysis. XRD analysis further revealed a face-centered cubic (fcc) structure, each one of which lies between that of pure fcc Pd (JCPDS no. 46-1043) and pure fcc Cu (JCPDS no. 85–1326), for the two TNPs and SNP (Supplementary Fig 1). The ultraviolet-visible-NIR spectra showed strong absorption in the NIR region (>800 nm) for both TNPs, with TNP-2 exhibiting higher absorption than TNP-1, consistent with the higher Pd ratio of TNP-2 (Fig. 1g). In contrast, significantly lower absorption was observed for SNP. These results strongly suggested that TNPs possess better potential as an NIR photothermal agent than SNP. To demonstrate this, we assessed temperature elevation profile of aqueous dispersions of CuPd nanoparticles upon NIR irradiation with 808 nm laser at a power density of 1 W cm^2^-1. Indeed, rapid temperature increase was observed, reaching 71 and 67 °C after 300 s irradiation, for TNP-2 and TNP-1, respectively (Fig. 1h). In contrast, the temperature of SNP dispersion increased to less than 45 °C after 300 s irradiation. These results indicated that TNPs had dramatically enhanced photothermal conversion efficiency as compared to SNP, a property that may be explained by the theory that the sharp-tip structure of TNPs could concentrate light at their tips and thus promote better local heat generation^40,41^.

### TNP-1 induced complete autophagy in a shape- and composition-dependent manner

**Fig. 2:**
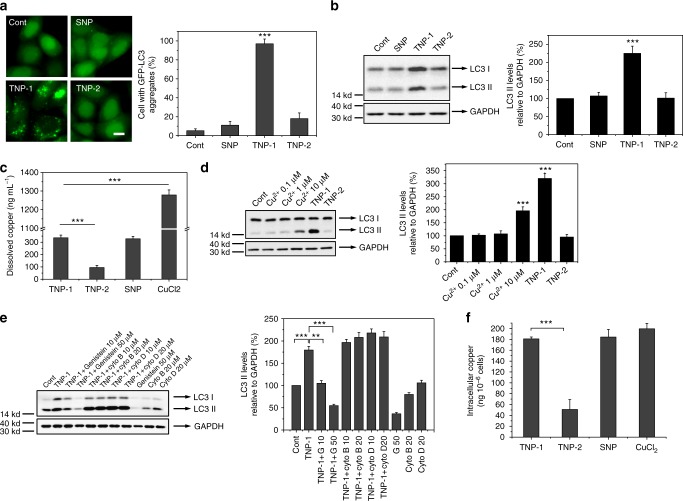
TNP-1 induced autophagy in a shape- and composition-dependent manner. **a** Fluorescent microscopy images of EGFP-LC3/HeLa cells treated with PBS (control) or 10 µg mL^−1^ of CuPd nanoparticles for 24 h. Scale bar, 10 µm. The right panel shows the quantified results for the percentage of cells containing at least 5 EGFP-LC3 dots. Mean ± s.e.m. *n* = 5. ***p < 0.001. Student’s *t*-test. **b** HeLa cells were treated with PBS (control) or 10 µg mL^−1^ of CuPd nanoparticles for 24 h and then subject to western blotting with anti-LC3 and GAPDH antibodies, with the densitometric analysis shown in right. Mean ± s.e.m. *n* = 3. ***p < 0.001. Student’s *t*-test. **c** ICP-MS assay of the free copper ions (Cu ^2+^) released from 10 µg mL^−1^ CuPd TNPs and SNP in DMEM after 24 h incubation. Mean ± s.e.m. *n* = 3. ***p < 0.001. Student’s *t*-test. **d** Western blotting of LC3 and GAPDH (served as loading control) in HeLa cells treated with varying concentrations of Cu ^2+^ or 10 µg mL^−1^ CuPd TNPs, and quantified by densitometric analysis relative to GAPDH. Mean ± s.e.m. *n* = 3. ***p < 0.001. Student’s *t*-test. **e** Western blotting of LC3 and GAPDH after HeLa cells were subject to the indicated treatment for 24 h. Concentrations used: TNP-1, 10 ug mL^−1^; Genistein, 10 μM, 50 μM; Cytochalasin B: 10 μM, 20 μM; Cytochalasin D: 10 μM, 20 μM. The right panel showed quantified results. Mean ± s.e.m. *n* = 3, **p < 0.01, ***p < 0.001. Student’s *t*-test. **f** HeLa cells exposed to 10 µg mL^−1^ CuPd TNPs, SNP or 1 μM Cu ^2+^ for 24 h. Intracellular copper were determined by ICP-MS. Mean ± s.e.m. *n* = 3. ***p < 0.001. Student’s *t*-test

We next assessed the autophagy-inducing activity of the two TNPs as well as SNP in EGFP/HeLa, a cell line that stably expressed enhanced green fluorescent protein-tagged LC3 (EGFP-LC3). EGFP-LC3 was evenly distributed in the untreated cells, but upon TNP-1 treatment appeared as bright punctate dots under fluorescent microscopy (Fig. 2a, with the quantified results shown on the right panel), indicating that TNP-1 induced autophagy. Consistent with the increased EGFP-LC3 dot formation, LC3 conversion, representing the endogenous soluble LC3-I protein converted to lipid bound LC3-II after attachment to the autophagosome membranes, was significantly elevated after TNP-1 treatment (Fig. 2b). The ability of TNP-1 to elicit autophagy was shape-dependent, as SNP, the SNPs that possess the same composition as that of TNP-1, failed to induce autophagy (Fig. 2a, b). The ability of TNP-1 to elicit autophagy was also composition-dependent, as TNP-2, the nanoparticles that exhibited almost identical shape as that of TNP-1 but had a lower Cu content, did not induce autophagy (Fig. 2a, b). Notably, nanomaterials with high Cu content have been reported to induce autophagy^32^, consistent with our results.

To assess the possibility that induction of autophagy by TNP-1 was due to the released Cu^2+^ ion, we conducted in vitro release assay. TNP-1 released similar amount of Cu^2+^ ion as SNP over a 24 h period (Fig. 2c), indicating that nanoparticle shape did not significantly affect Cu^2+^ ion release. Much lower amount of Cu^2+^ ion was released by TNP-2, consistent with its low Cu content. As SNP did not induce autophagy, this result implies that autophagy induction by TNP-1 was not mediated by the released Cu^2+^ ion. As additional evidence, 1 μM Cu^2+^ ion, representing approximately fourfold concentration as that of Cu^2+^ ion released from TNP-1 over a 24 h period, failed to elicit autophagy (Fig. 2d).

To assess the role of endocytosis for the autophagy-inducing effect, we co-treated HeLa cells with TNP-1 and endocytic inhibitors. Genistein, an inhibitor of caveolae-mediated endocytosis, significantly inhibited LC3 conversion induced by TNP-1 (Fig. 2e), indicating that cell internalization is required for TNP-1’s autophagy-inducing activity. In contrast, cytochalasin B and D, inhibitors of clathrin-mediated endocytosis, were ineffective. To assess the possibility that induction of autophagy by TNP-1 but not SNP was due to difference in the amount of internalized nanoparticle, we measured the level of intracellular Cu after incubation of nanoparticles with cells for 24 h (Fig. 2f). The level of intracellular Cu, which presumably included both internalized Cu and Cu stably associated with the cell surface, was similar for cells treated with TNP-1 and SNP, indicating that nanoparticle shape did not significantly affect nanoparticle internalization and also strongly suggested that the ability of TNP-1 to induce autophagy was not due to increased cellular internalization.

**Fig. 3:**
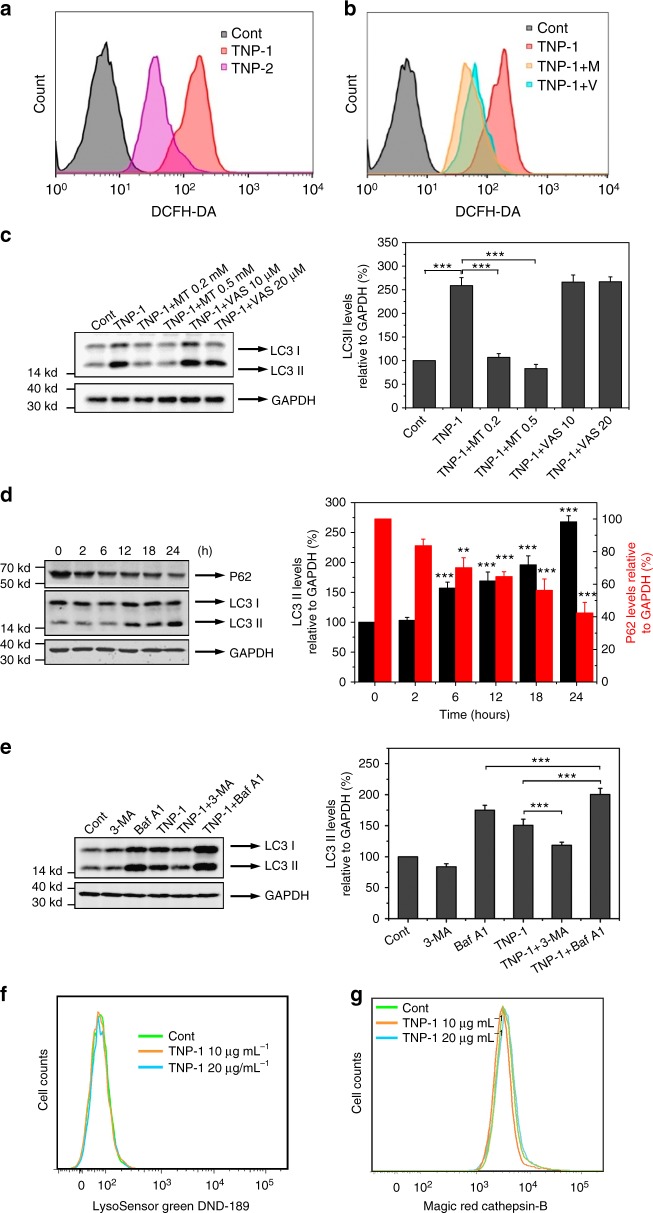
TNP-1 induced complete autophagy in a ROS-dependent fashion. **a** Intracellular ROS detected with DCFH-DA and analyzed by FACS after treatment with PBS (control) or TNPs (10 µg mL^−1^) for 24 h. **b** FACS analysis of intracellular ROS after HeLa cells were treated with PBS (control), TNP-1 (10 µg mL^−1^), or TNP-1 in the presence of MitoTempo (M; 0.5 mM) or VAS 2870 (V; 20 μM) for 24 h. **c** Western blotting of HeLa cells with LC3 and GAPDH antibodies after the indicated treatment for 24 h. The right panel showed quantified results. MT, MitoTemper; VAS, VAS 2870. Mean ± s.e.m. *n* = 3. ***p < 0.001. Student’s *t*-test. **d** Western blotting of p62, LC3 and GAPDH in HeLa cells after treatment with TNP-1 (10 µg mL^−1^) for the various times. The right panel showed quantified results. Mean ± s.e.m. *n* = 3. ***p < 0.001. Student’s *t*-test. **e** Western blotting of LC3 and GAPDH after HeLa cells were subject to the indicated treatment for 24 h. Concentrations used: TNP-1, 10 ug mL^−1^; 3-MA, 2.5 mM; bafilomycin A1 (BFA A1): 400 nM. The right panel showed quantified results. Mean ± s.e.m. *n* = 3. ***p < 0.001. Student’s *t*-test. **f** FACS analysis of HeLa cells after treatment with PBS (control), 10 or 20 µg mL^−1^ TNP-1 for 24 h followed by staining with 1 µmol L^−1^ LysoSensor Green DND-189 for 30 min. **g** HeLa cells were treated with PBS (control), 10 or 20 µg mL^−1^ TNP-1 for 24 h. Cathepsin activity was then detected with the Magic Red Cathepsin-B Assay and analyzed by FACS

To further investigate the mechanism underlying the drastically different autophagy behavior of TNP-1 versus TNP-2, we assessed intracellular reactive oxygen species (ROS), which are known to be induced by a variety of nanoparticles. TNP-1 induced much higher elevation of intracellular ROS than TNP-2 as revealed by FACS analysis (Fig. 3a). Both MitoTempo, an inhibitor of mitochondria ROS, and VAS 2870, an inhibitor of NADPH oxidase, reduced TNP-1-elicited ROS (Fig. 3b), but only MitoTempo significantly inhibited TNP-1-elicited autophagy (Fig. 3c). On the other hand, neither 3-MA nor CQ affected the high-level ROS elicited by TNP-1 (Supplementary Fig 2), consistent with the notion that ROS was upstream of autophagy. These results also indicated that mitochondria-generated ROS is critically important for TNP-1-induced autophagy and suggested that the difference in inducing mitochondrial ROS production may account for the differential autophagy-inducing activity of TNP-1 versus TNP-2.

Additional experiments were conducted to further characterize the autophagy induced by TNP-1 in HeLa cells. The nanoparticles induced LC3-II conversion in a time and dose-dependent fashion, with the maximal effect observed at 24 h for 10 µg mL^−1^ of TNP-1 (Fig. 3d and Supplementary Fig 3, respectively). Time-course study also revealed a parallel decrease in the level of p62, a common substrate of autophagy (Fig. 3d, with the densitometric quantification results shown on the right panel), while no such decrease was observed after TNP-2 treatment (Supplementary Fig 4). 3-Methyladenine (3-MA), an autophagy inhibitor that blocks the formation of autophagosomes through inhibition of the class III phosphoinositol 3-kinase pathway, effectively inhibited TNP-1-induced LC3-II conversion. Meanwhile, TNP-1 treatment led to an increase, while co-treatment with bafilomycin A1, an inhibitor of autolysosome degradation, caused a further increase, in the level of LC3-II (Fig. 3e). These results indicated that the autophagy induced by TNP-1 exhibited normal flux. Furthermore, flow cytometry analysis using Lysosensor Green DND-189, an acidotropic dye that accumulates in acidic organelles and exhibits fluorescence intensity proportional to acidity, revealed that TNP-1 treatment did not significantly alter lysosomal acidity (Fig. 3f). Lysosomal enzyme cathepsin B release assay also revealed that TNP-1 did not disrupt lysosomal function (Fig. 3g), in agreement with the autophagic flux assay results described above.

Taken together, the above results demonstrated that TNP-1 induced, through enhanced mitochondrial ROS production, complete autophagy with no disruption of lysosomal function.

### Autophagy induced by TNP-1 promoted cancer cell survival

**Fig. 4:**
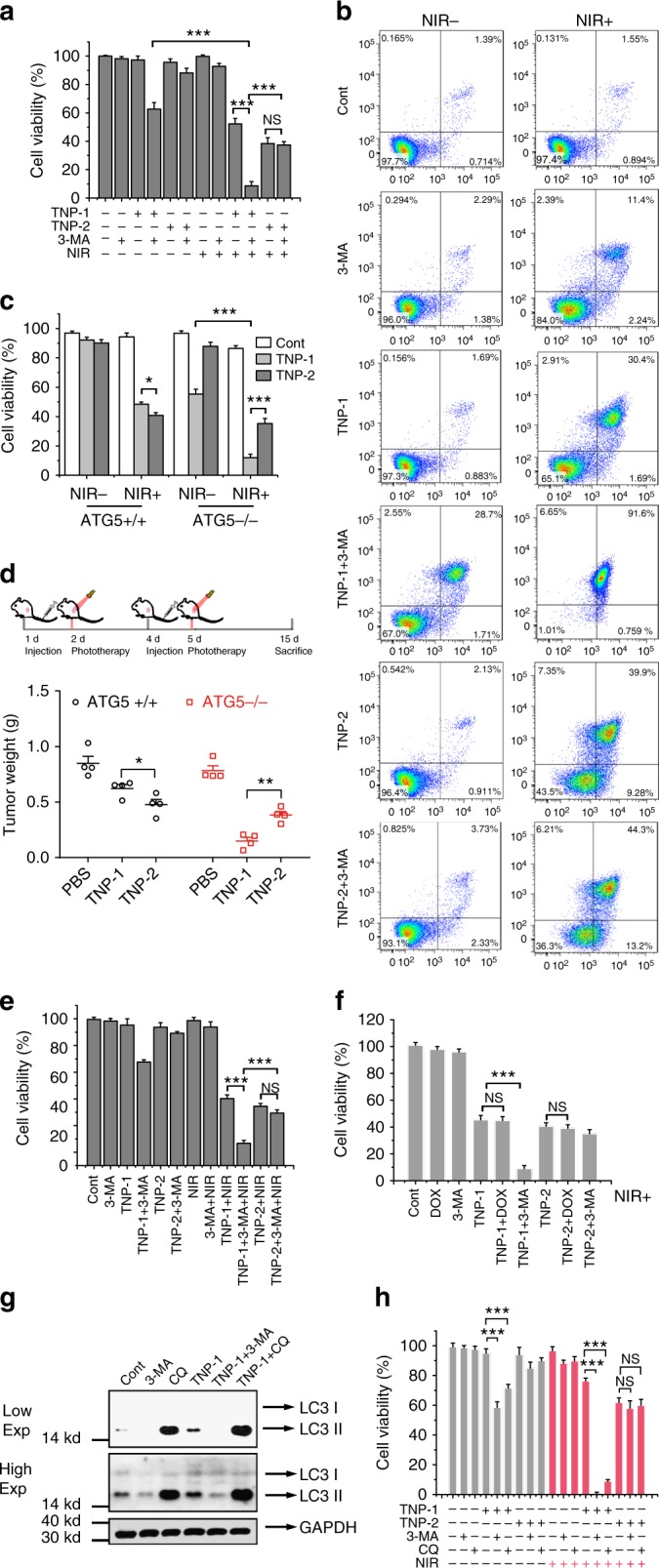
Autophagy induced by CuPd TNP-1 promoted cell survival. **a,** **b** Cell viability (**a**) and Annexin-V/PI assay (**b**) of HeLa cells after treatment for 24 h with the indicated combination. TNP-1 or TNP-2: 10 μg mL^-1^; 3-MA: 2.5 mM; NIR irradiation: 1 W cm^2^^-1, 3 min. FACS analysis in **b** showed the relative percentage of live (lower-left quadrant), early apoptotic (lower-right quadrant) and late apoptotic/necrotic (upper-right quadrant) cells. Mean ± s.e.m. *n* = 5, ***p < 0.001. Student’s t-test. **c** Cell viability of wild-type (*Atg5* + / + ) and *Atg5* knockout (*Atg5*-/-) HeLa cells after treatment with PBS (control), 10 μg mL TNP-1 or 10 μg mL^−1^ TNP-2 for 24 h in the presence of absence of NIR irradiation for 3 min. Mean ± s.e.m. *n* = 5, ***p < 0.001. Student’s *t*-test. **d** Top panel: the 15-day PTT regimen, with the mice receiving tail vein injection (PBS or 1.5 mg kg^−1^ of TNP-1 or TNP-2) on day 1 and 4 and PTT treatment on the tumor on day 2 and 5. Lower panel: the tumor weight on day 15. Mean ± s.e.m. *n* = 4, *p < 0.05, **p < 0.01, ***p < 0.001. Student’s *t*-test. **e,** **f** Cell viability of 4T1 (**e**) and MCF-7/MDR (**f**) cells after treatment for 24 h with the indicated combination. TNP-1 or TNP-2: 10 μg mL^−1^; 3-MA: 2.5 mM; Dox, 25 mM; NIR irradiation: 1 W cm^2^^-1, 3 min. Mean ± s.e.m. *n* = 5, ***p < 0.001. Student’s *t*-test. **g** Western blotting of breast cancer patient-derived tumor cells after treatment with PBS (control) or 10 μg mL^−1^ CuPd TNPs for 24 h. The right panel showed quantified results. Mean ± s.e.m. *n* = 3. ***p < 0.001. Student’s *t*-test. **h** Cell viability of breast cancer patient-derived tumor cells after treatment for 24 h with the indicated combination. TNP-1 or TNP-2: 10 μg mL^−1^; 3-MA: 2.5 mM; CQ, 25 mM; NIR irradiation: 1 W cm^2^^-1, 3 min. Mean ± s.e.m. *n* = 5, ***p < 0.001. Student’s *t*-test

As described above, the induced autophagy could have either pro-death or pro-survival impact on the cancer cell. To determine which was the case, we assessed cell viability in HeLa, using the well-established MTT assay. 3-MA or NIR irradiation alone had no effect, while their combination showed a small and insignificant decrease, on cell viability (Fig. 4a). In the absence of NIR irradiation, TNP-1 elicited minimal change in cell viability. On the other hand, co-treatment of TNP-1 with 3-MA resulted in a 37% decrease in cell viability, indicating that TNP-1 induced pro-survival autophagy. These results also indicated that the pro-survival autophagy was sufficient to block the potential death-evoking effect of the high-level ROS elicited by TNP-1 (Fig. 3a). In agreement, the ROS scavenger N-acetyl cysteine was able to effectively inhibit cell death occurred after TNP-1 plus 3-MA treatment (Supplementary Fig 5). In comparison, 3-MA caused a much smaller cell viability reduction when co-treated with TNP-2, an ineffective autophagy inducer. Irradiation with 1 W cm^2^^-1 NIR light for 3 min, a treatment with relatively low power and short duration as compared to treatment conditions for other Pd- or Cu-based PTT nanomaterials, enhanced the cytotoxicity of TNP-2 and TNP-1. The enhancement effect was somewhat higher for TNP-2 than for TNP-1, consistent with the higher photothermal conversion efficiency of TNP-2. Importantly, NIR irradiation plus 3-MA had a remarkable synergistic effect on TNP-1-elicited cytotoxicity, resulting in 88% reduction in HeLa cell viability, which was significantly higher than the reduction in cell viability after TNP-1 plus irradiation treatment. In contrast, 3-MA did not further decrease cell viability in HeLa cells treated with TNP-2 and NIR irradiation. Notably, autophagy inhibition with 3-MA enhanced the cytotoxicity of TNP-1-mediated PTT to a level unattainable under TNP-2-mediated PTT either with or without 3-MA (less than 65% viability reduction).

A different assay, measuring cell death using Annexin V/PI staining, revealed the same conclusions (Fig. 4b and summarized in Supplementary Table 1). 3-MA significantly increased TNP-1-elicited, but only marginally increased TNP-2-elicited, cell death. NIR irradiation enhanced cell death elicited by both TNPs, while the addition of 3-MA further increased cell death for TNP-1 but not TNP-2. Notably again, autophagy inhibition helped TNP-1-mediated PTT to achieve a level of cell death unattainable under TNP-2-mediated PTT, as near complete killing was achieved with the combined treatment of TNP-1, irradiation and 3-MA (>92%, with the lower-right and the upper-right quadrants combined, which included both early apoptotic and late apoptotic/necrotic cells), while much less killing (55%) was observed for the combined treatment of TNP-2, irradiation and 3-MA.

To gain further proof for the role of autophagy in cell fate determination, we knocked out *Atg5*, an autophagy-essential gene, in HeLa cells with the aid of CRISPR/Cas9 technology. As would be expected, *Atg5* knockout led to reduced LC3-II conversion, indicative of decreased autophagy, after TNP-1 treatment (Supplementary Fig 6). Importantly, *Atg5* knockout significantly increased cytotoxicity elicited by TNP-1 but not by TNP-2, either in the absence or presence of NIR irradiation, as shown by both the viability assay (Fig. 4c) and Annexin V/PI staining assay (Supplementary Fig 7 and summarized in Supplementary Table 2).

We also assessed the impact of *Atg5* knockout on TNP-mediated PTT in tumors of HeLa subcutaneously implanted on NOD/SCID mice, using a 15-day treatment regimen (Fig. 4d, top panel). Tumors were excised on day 15, photographed (Supplementary Fig 8) and weighed (Fig. 4d, bottom panel). TNP-2 + NIR had better therapeutic efficacy than TNP-1 + NIR in the wild-type tumors, in agreement with the higher photothermal efficiency of TNP-2. However, in the *Atg5* knockout tumors, the opposite was true, with the tumors treated by TNP-1 + NIR being significantly smaller than the tumors treated by TNP-2 + NIR, indicating that loss of autophagy enhanced the therapeutic effect of TNP-1-mediated PTT. There was also a statistically-significant difference in therapeutic efficacy between the wild-type and knockout tumors for TNP-1 + NIR treatment, as measured by the tumor reduction ratio (26.72% for wild-type tumor and 80.92% for *Atg5* knockout tumor, *p* < 0.001, Student’s *t*-test), which was defined by the formula (Tumor weight after PBS + NIR treatment − Tumor weight after TNP-1 + NIR treatment)/(Tumor weight after PBS + NIR treatment). On the other hand, no statistically-significant difference in this ratio was observed between the wild-type and knockout tumors for TNP-2 + NIR treatment (43.82% for wild-type tumor and 47.08% for *Atg5* knockout tumor, *p* > 0.05, Student’s *t*-test). These results agreed well with the in vitro studies described above and provided convincing evidence that the authphagy induced by TNP-1 played a cyto-protective role for HeLa cells.

We next investigated the potential autophagy-modulating effect of TNP nanoparticles in the triple-negative (ER-, PR-, HER2-) murine mammary carcinoma cell line 4T1, which was known to be a difficult-to-treat cancer, and multidrug-resistant human breast carcinoma cell line MCF7/MDR, which showed minimal death after treatment with 20 μM doxorubicin for 24 h (Supplementary Fig 9). Similar to the situation in HeLa, TNP-1 induced autophagy, while TNP-2 and SNP did not, in these two cell lines (Supplementary Fig 10a). 3-MA effectively inhibited TNP-1-elicited autophagy in both cell lines (Supplementary Fig 10b). Co-treatment of TNP-1, but not TNP-2, with 3-MA resulted in a significant decrease in cell viability (32% in 4T1 and 35% in MCF7/MDR; Fig. 4e, f and Supplementary Fig 11, respectively). NIR irradiation plus 3-MA had a synergistic effect on TNP-1-elicited cytotoxicity, resulting in cell viability reduction of 92% in 4T1 and 85% in MCF7/MDR. In contrast, less than 65% reduction in 4T1 and MCF7/MDR cell viability was achieved by the combination of NIR and TNP-2, regardless whether 3-MA was added. Similar results were obtained with the Annexin V/PI staining assay. The combined treatment of TNP-1, irradiation and 3-MA led to near complete killing (95% in 4T1 and 92% in MCF7/MDR; Supplementary Fig 12 and 13, respectively), while much less killing (56% in 4T1 and 63% in MCF7/MDR) was observed for the combined treatment of TNP-2, irradiation and 3-MA. Both assays also revealed that Dox did not increase MCF7/MDR cell death elicited by TNPs (either TNP-1 or TNP-2) plus NIR irradiation, suggesting that chemo-PTT using Dox would fail in this drug-resistant cell line.

To gain a preliminary indication on whether TNP-1 would induce cytoprotective autophagy in patient-derived tumors, we isolated primary cancer cells from the freshly excised tissues of a breast cancer and a gastric cancer patient. TNP-1, but not TNP-2, induced apparent autophagy in the cells derived from the breast cancer patient (Fig. 4g). Furthermore, both CQ and 3-MA dramatically enhanced TNP-1-mediated, but not TNP-2-mediated, photothermal killing in these cells (Fig. 4h). Notably, these cells exhibited a drug-resistant profile, as this patient has failed three rounds of neoadjuvant chemotherapy attempts previously. In comparison, TNP-1 induced marginal autophagy in the cells derived from the gastric cancer patient (Supplementary Fig 14), with CQ and 3-MA showing much reduced but still significant enhancement on TNP-1-mediated photothermal cell killing (Supplementary Fig 15). These results indicated that the ability of TNP-1 to induce cytoprotective autophagy is tumor-specific, and that the level of induced autophagy correlated with the extent of enhancement on TNP-1-mediated photothermal cell killing.

### CQ and 3-MA significantly enhanced anti-cancer efficacy of TNP-1-mediated PTT in the 4T1 breast cancer model

**Fig. 5:**
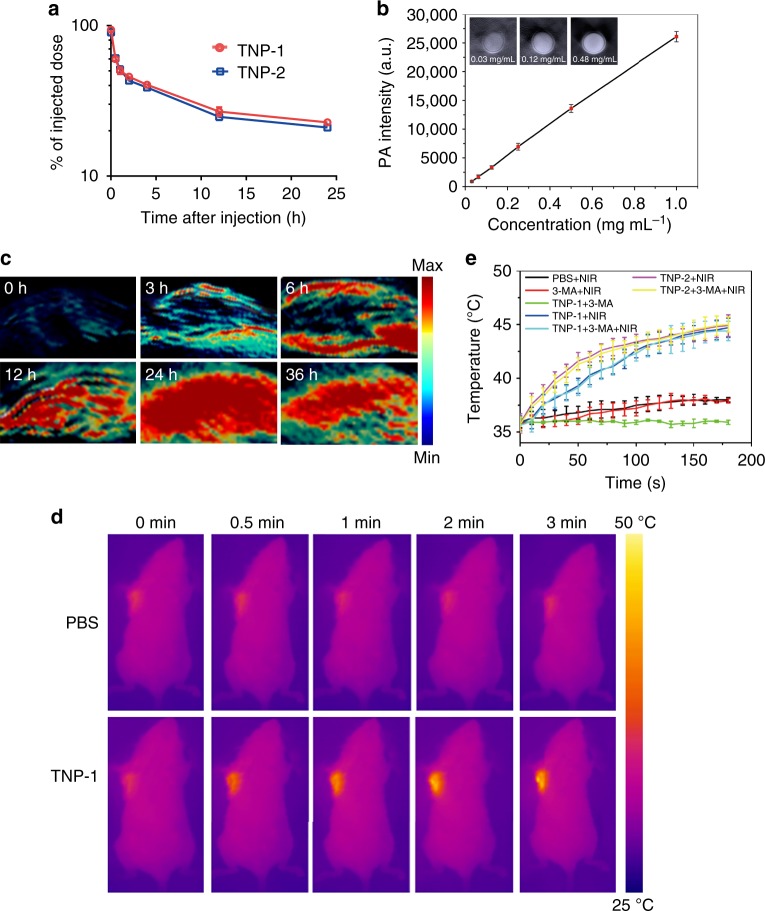
Pharmacokinetics and imaging properties of TNP-1. **a** Pharmacokinetic profile of TNP-1 and TNP-2. Mean ± s.e.m. *n* = 3. Student’s t-test. **b** Photoacoustic images and photoacoustic intensity of TNP-1 of different concentrations in deionized water. **c** In vivo photoacoustic images of the 4T1 breast tumor at different time points after intravenous injection of PBS (control) and TNP-1. **d** The thermographs of mice recorded while NIR irradiation for the various times, 24 h after the injection of PBS or TNP-1. **e** The tumor-site temperature changes recorded while NIR irradiation for the various times, 24 h after the indicated PTT treatment

We started tumor therapeutic study by conducting preliminary evaluation on the pharmacokinetics and bio-safety of TNP nanoparticles. TNP-1 and TNP-2 had a nearly identical pharmacokinetic profile (Fig. 5a), exhibiting similar pharmacokinetic parameters such as T_1/2_, AUC, C_MAX_, and MRT (Supplementary Table 3). Analysis of key serum biochemical markers for liver (alanine aminotransferase and aspartate transaminase), and kidney (blood urea nitrogen and serum creatinine) toxicity revealed no statistically significant difference in any of these markers between nanoparticle (TNP-1 or TNP-2)-treated and non-treated mice (Supplementary Fig 16). We have also conducted HE staining on major organs (liver, spleen, kidney, lung, heart, and brain) of mice and found no apparent tissue damage in any of these organs after treatment with TNP-1 or TNP-2 (Supplementary Fig 17).

We next proceeded to evaluate the anti-tumor efficacy of TNP-mediated PTT in an orthotopic 4T1 breast cancer model. To establish the best timing for initiating PTT treatment, we took advantage of the multi-spectral optoacoustic tomography (MSOT) property of CuPd TNPs. Phantom studies revealed that the mean pixel intensity obtained from MSOT increased linearly with the increasing concentrations of TNP-1 (Fig. 5b), establishing a linear relationship between the intensity of the MSOT images and the relative quantity of TNP-1. We next assessed the MSOT imaging property in vivo after intravenous injection of TNP-1 at a dose of 1.5 mg kg^−1^. The photoacoustic signals at the tumor site increased significantly at 3 h and reached maximum at 24 h post injection (Fig. 5c). Consistent with this, irradiation of the tumor site at the various times after TNP-1 administration revealed time-dependent temperature elevation, indicating increasing nanoparticle accumulation, that reached maximum at 24 h (Fig. 5d). We thus chose the 24 h post nanoparticle delivery as the time point for the PTT treatment, and we carefully controlled the irradiation time to ensure, with the aid of camera, that the temperature of the tumor site was between 44 and 45 °C (Fig. 5e). This temperature was achieved within 3 min of irradiation for the mice injected with TNP-1 or TNP-2. In contrast, the tumor site temperature of the mice injected with PBS showed an increase of less than 2 °C (<39 °C) under the same treatment.

**Fig. 6:**
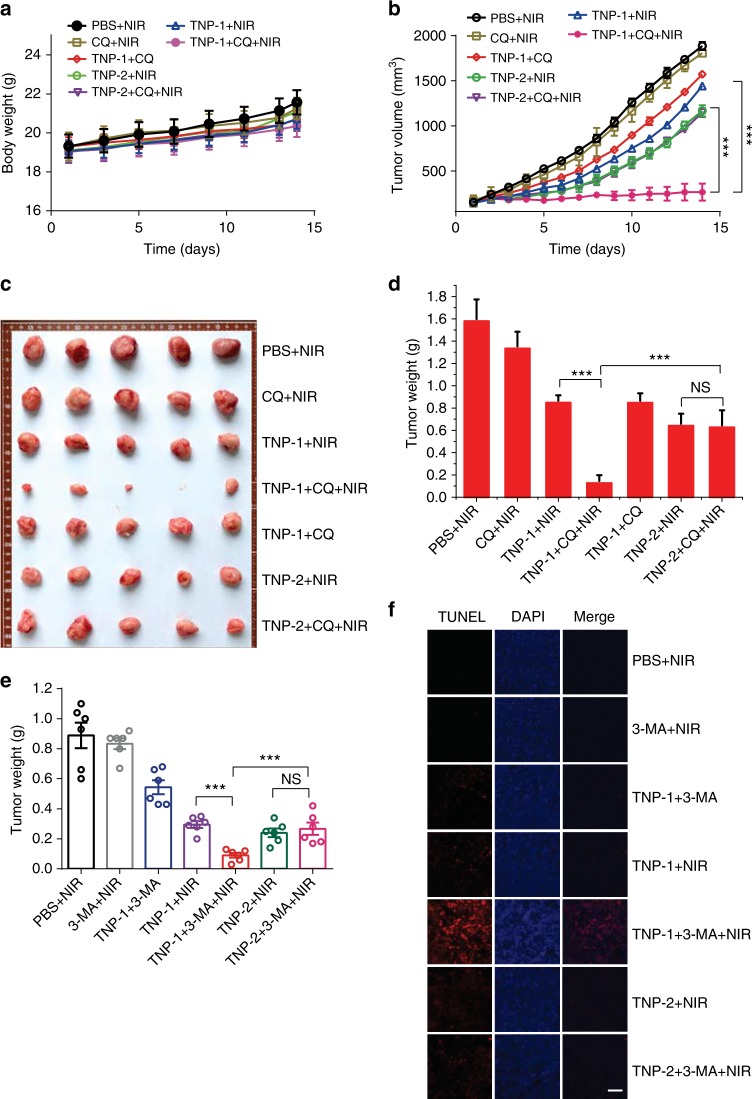
Autophagy inhibitors significantly enhanced anti-cancer efficacy of TNP-1-mediated PTT in the 4T1 model. **a,** **b** The body weight (**a**) and tumor volume (**b**) changes of mice in the various treatment groups during the 15-day therapeutic period. Dosing used: TNP-1 or TNP-2, 1.5 mg kg^−1^; CQ, 25 mg kg^−1^; 3-MA, 100 μmol kg^−1^. Mean ± s.e.m. *n* = 5. ***p < 0.001. Student’s *t*-test. **c** Photographs of the tumors excised on day 15. **d** Tumor weight of mice on day 15 in the various treatment groups. Mean ± s.e.m. *n* = 5. **p < 0.01, ***p < 0.001. Student’s *t*-test. **e** Tumor weight of mice in the various treatment groups after a 7-day PTT treatment regimen in a different study. Mean ± s.e.m. *n* = 6. ***p < 0.001. Student’s *t*-test. **f** TUNEL staining (red) of sections from tumor in (**e**) was performed to show apoptotic cells. Nucleus were stained with DAPI (blue). Scale bar, 50 μm

Tumor PTT was conducted using the same 15-day treatment regimen (Fig. 4d), starting when the tumor volumes reached about 100 nm^3^. No significant difference was observed for the body weight change during the course of therapy among different treatment groups (Fig. 6a). PTT treatment mediated by either TNP-1 or TNP-2 significantly inhibited tumor growth, with TNP-2 exhibiting a slightly better effect, as indicated by the change in tumor volume (Fig. 6b). Co-treatment with CQ, an autophagy inhibitor currently used in clinical trials, further reduced the tumor volume for the TNP-1 + NIR, but not the TNP-2 + NIR, group. On day 15 the tumors from each group were harvested and photographed (Fig. 6c). Compared to the control groups (PBS + NIR and CQ + NIR), the PTT groups (TNP-1 + NIR and TNP-2 + NIR) exhibited significantly smaller tumor, with no statistical difference between the two PTT groups (Fig. 6d). Adding CQ further enhanced the anti-cancer efficacy for the TNP-1-mediated, but not the TNP-2-mediated, PTT, as the tumor weight of the TNP-1 + NIR + CQ group decreased by 92% compared to the PBS + NIR group, while the corresponding number was only 59% for the TNP-2 + NIR + CQ group, revealing a statistically significant difference between the two chemo-PTT groups (*p* < 0.001, Student’s *t*-test).

In a separate study, 3-MA instead of CQ was used as the autophagy inhibitor. At the end of the 7-day treatment regimen, the tumors from each group were harvested and photographed (Supplementary Fig 18). Compared to the control groups (PBS + NIR and 3-MA + NIR), the PTT groups (TNP-1 + NIR and TNP-2 + NIR) exhibited significantly smaller tumor, with no statistical difference between the two PTT groups (Fig. 6e). Similar to CQ, 3-MA further enhanced the anti-cancer efficacy for TNP-1- but not TNP-2-mediated PTT, as the tumor weight of the TNP-1 + NIR + 3-MA group decreased by 91% compared to the PBS + NIR group, while the corresponding number was only 67% for the TNP-2 + NIR + 3-MA group. TUNEL assay showed many more apoptotic cells in the mice treated with TNP-1 + NIR + 3-MA than in the mice in the other PTT treatment groups, while few apoptotic cells were observed in the mice treated with either PBS + NIR or 3-MA + NIR (Fig. 6f). As would be expected, the level of autophagy in the tumor tissue was significantly elevated after TNP-1 treatment, and this elevated autophagy was effectively inhibited by 3-MA (Supplementary Fig 19).

### CQ and 3-MA significantly enhanced anti-cancer efficacy of TNP-1-mediated PTT in the drug-resistant MCF7/MDR breast cancer model

**Fig. 7:**
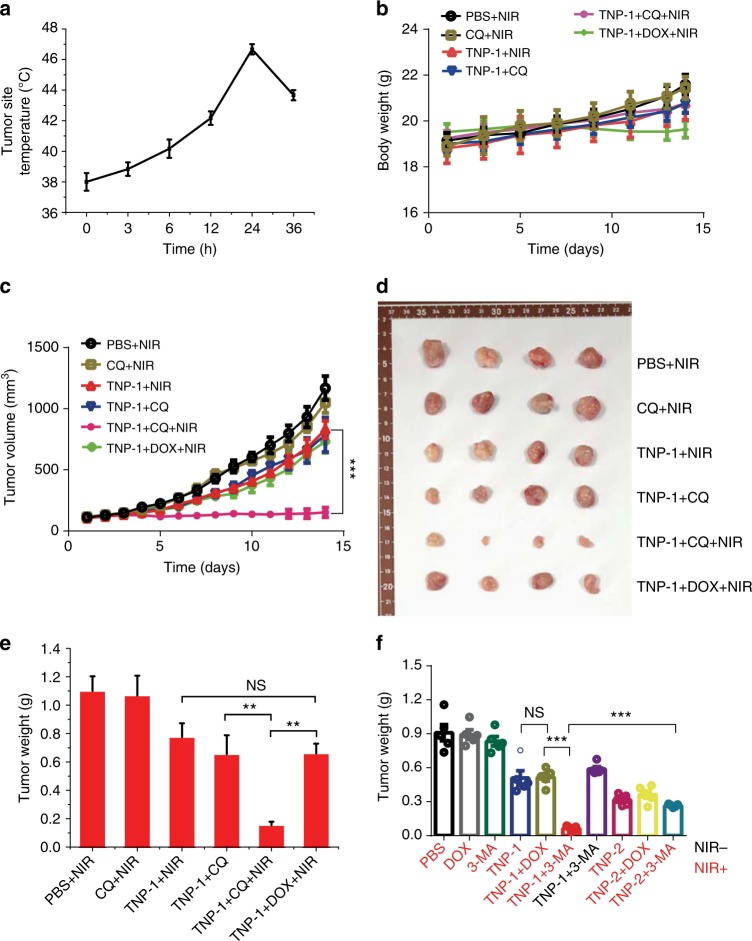
Autophagy inhibitors significantly enhanced anti-cancer efficacy of TNP-1-mediated PTT in the MCF7/MDR model. **a** Tumor-site temperature recorded after three minute of NIR irradiation, at the various time points after the intravenous injection of TNP-1. Mean ± s.e.m. *n* = 3. Student’s *t*-test. **b,** **c** The body weight (**b**) and tumor volume (**c**) changes of mice in the various treatment groups during the 15-day therapeutic period. Dosing used: TNP-1 or TNP-2, 1.5 mg kg^-1^; CQ, 25 mg kg^−1^; Dox, 15 mg kg^−1^. Mean ± s.e.m. *n* = 5. ***p < 0.001. Student’s *t*-test. **d** Photographs of the tumors excised on day 15. **e** Tumor weight of mice on day 15 in the various treatment groups. Mean ± s.e.m. *n* = 5. **p < 0.01, ***p < 0.001. Student’s *t*-test. **f** Tumor weight of mice in the various treatment groups after a 7-day PTT treatment regimen in a different study.Mean ± s.e.m.*n* = 6. ***p < 0.001. Student’s *t*-test

To assess the anti-tumor efficacy of TNPs-mediated PTT for drug-resistant cancer, we established an MCF7/MDR breast cancer model in NOD/SCID mice. Photothermal imaging revealed that the maximum nanoparticle accumulation was achieved 24 h post TNPs injection (Fig. 7a). We thus chose this time point to conduct the PTT treatment, using the same 15-day treatment regimen (Fig. 4d). No significant difference was observed for the body weight change during the course of therapy among different treatment groups, although the mice in the TNP-1 + Dox + NIR group exhibited somewhat lower body weight, presumably due to the toxicity of Dox (Fig. 7b). PTT treatment mediated by TNP-1 significantly inhibited tumor growth, with CQ but not Dox further reducing the tumor volume under the TNP-1-mediated PTT (Fig. 7c). Tumor weight at the end of the experimental period agreed well with the tumor volume results (Fig. 7d, e). Adding Dox did not further enhance PTT efficacy for TNP-1, indicating that conventional chemo-PTT would fail in these drug-resistant cancer cells. Importantly, dramatically reduced tumor size was observed with the combination of TNP-1, NIR and CQ, with the tumor weight decreased by 85.93% as compared to the control group (PBS + NIR).

The same conclusions were reached in a separate study using 3-MA as the autophagy inhibitor. At the end of the 7-day treatment regimen, the tumors were harvested and photographed (Supplementary Fig 20). Dox was ineffective in this drug-resistant tumor model, with the mice exhibiting similar size as the control groups (PBS + NIR and 3-MA + NIR). PTT treatments (TNP-2 + NIR and TNP-1 + NIR) significantly reduced tumor weight, with no statistical difference between the two PTT groups (Fig. 7f). Adding Dox did not further enhance PTT efficacy for either TNP-2 or TNP-1. In contrast, dramatically reduced tumor size was observed with the combination of TNP-1, NIR and 3-MA, with the tumor weight decreased by 93% as compared to the control group (PBS + NIR). In comparison, only 64% decrease in tumor weight was observed for the combination of TNP-2, NIR and 3-MA. The TUNEL assay revealed the number of apoptotic cells in opposite correlation with the tumor volume, with the most apoptotic cells seen in the combination treatment of TNP-1, NIR, and 3-MA (Supplementary Fig 21)

## Discussion

Induction of autophagy is a common, but not always, response of cells upon exposure to nanomaterials^32,42,43^. A great variety of nanomaterials have been shown to induce autophagy in cancerous as well as normal cells. Chemical composition, size, shape and surface properties of the nanomaterial all play important roles in determining whether a nanomaterial will induce autophagy in a given cell under given circumstances. In this work we demonstrated that TNP-1 induced autophagy in a shape- and composition-dependent manner, as neither SNP, which had the same composition but different shape, nor TNP-2, which had same shape but different composition, induced autophagy under the same circumstances. Enhanced production of intracellular ROS, particularly ROS generated in mitochondrial, but not the extent of nanoparticle internalization or the level of nanoparticle-released Cu2+ ion, was found to be the underlying mechanism for the distinctive ability of TNP-1 to induce autophagy. Furthermore, we demonstrated that autophagy induced by TNP-1 was pro-survival, as inhibition of autophagy enhanced cancer cell killing elicited by TNP-1. The fact that the autophagy induced by TNP-1 exhibited normal flux without impairment of lysosomal function was consistent with this pro-survival role.

Combining PTT with chemotherapy, or chemo-PTT, is a powerful approach in cancer therapy, as the two treatment modalities kill cancer cells through different mechanisms and oftentimes complement each other well for achieving complete eradication of tumor. However, cancer cells are prone to acquire resistance to chemotherapeutic agents after repeated exposure, an issue that is becoming more and more prudent in clinical practice today. Thus conventional chemotherapeutic drugs have limited utility for chemo-PTT of drug-resistant cancer. Novel chemotherapeutic agents, operating through mechanisms distinct from those of traditional chemotherapeutics, would be invaluable in helping overcome this problem. In this work we demonstrated, as a proof-of-concept, that chemical inhibitors of autophagy may replace traditional chemotherapeutics to accomplish nanomaterial-enabled chemo-PTT in certain situations. Specifically, this chemo-PTT strategy would work if the PTT-ready nanomaterial induces pro-survival autophagy. Such material may be engineered, as we did here by tuning the composition and shape of CuPd alloy nanoparticles. Importantly, inhibition of this pro-survival autophagy renders the cancer cells more susceptible to PTT-mediated killing, thus achieving synergistically-enhanced anti-cancer efficacy that is unattainable with another nanomaterial having the similar photothermal efficiency but no autophagy-inducing activity. The exact mechanism underlying this remarkable phenomenon remains to be determined.

A couple of other points about the CuPd alloy TNPs we synthesized in this work are noteworthy. One is their outstanding photothermal conversion efficiency. High photothermal conversion efficiency is extremely desirable in nanomaterial-facilitated PTT, as it would enable effective PTT under relatively low dosing, which may be critically important for minimizing nanomaterial-elicited toxicity. High photothermal conversion efficiency would also enable PTT to operate in a deep tissue, where light penetration is limited. TNP-1 we synthesized exhibited superior photothermal property, as the degree of temperature elevation required for PTT was easily achievable with a combination of low nanoparticle concentration, low laser power, and short duration of irradiation. The unique tetrapod structure of these nanoparticles was a key factor, as the spherical CuPd alloy nanoparticles (SNP) exhibited a much poorer photothermal response than TNP-1, even though the two nanoparticles had the same composition. We attributed this finding to the theory that the special sharp-tip structure of TNPs could concentrate light at their tips and thus promote better local heat generation than the spherical CuPd NPs. The other useful feature of TNP-1 was their capability for MSOT imaging, an emerging modality that brings great promise to overcome the obstacles encountered in pure optical imaging. NIR-assisted MSOT is noninvasive and can give both anatomical and functional information with deep tissue penetration and high spatial resolution. In this work we utilized MSOT imaging to visualize nanoparticle accumulation in the tumor tissue and to establish the timing for initiating PTT treatment. In clinics, this feature may also enable imaging-guided PTT, in which the MSOT images are used to guide laser irradiation and achieve precise tumor eradication, thus minimizing photothermal damage to normal cells. In short, TNP-1 represents a superior type of “one fits all” theranostic nanoparticles and is ideally suited for autophagy-inspired chemo-PTT of difficult-to-treat and drug-resistant cancer

## Methods

### Materials and antibodies

Ultrapure water (pH 6.7; Milli-Q, Bedford, MA) was used in all situations throughout the experiments. Sodium tetrachloropalladate (II) (Na_2_PdCl_4_), decylamine (DA), 3-Methyladenine (M9281), bafilomycin A1 (B1793), Chloroquine (C6628), MitoTEMPO (SML0737) and VAS2870 (SML0273) were purchased from Sigma-Aldrich Co., LLC. (Shanghai, China). Copper (I) chloride (CuCl), poly(vinyl pyrrolidone) (PVP, Mw≈30000), glucose (α or β form), N,N-dimethylformamide (DMF), and ethanol (C_2_H_6_O) were purchased from Sinopharm Chemical Reagent Co. Lid. (Shanghai, China). All chemicals were used as received without further purification. Anti-LC3 antibody (NB100–2220, 1:2000 dilution) were purchased from Novus Biologicals. Anti-GAPDH antibodies (AB9132, 1:10,000 dilution) were from Chemicon.,and anti-ATG5 antibody (sc-133158, 1:500 dilution) were from Santa Cruz Biotechnology. HRP-conjugated anti-rabbit antibody (W4011, 1:10,000 dilution) and HRP-conjugated anti-mouse antibody (W4021, 1:10,000 dilution) were purchased from Promega. The HeLa, 4T1, MCF7 cell lines were purchased from ATCC. MCF-7/MDR (kindly provided by Dr Jun Wang at South China University of Technology) were maintained in the DMEM medium containing doxorubicin.

### Synthesis of CuPd tetrapods

In a typical synthesis of Cu-rich CuPd tetrapods (CuPd TNP-1), 3.5 mg of Na2PdCl4, 5.7 mg of CuCl, 300 mg of PVP, 100 μL of DA, and 250 mg of glucose were dissolved in 5 mL of DMF in a 20-mL vial. After the vial had been capped, the vial was transferred into an oil bath and heated at 80 °C under vigorous magnetic stirring for 2 h. The obtained CuPd nanocrystals were collected by centrifugation and washed several times with deionized water and ethanol to remove the excess PVP, DA, and glucose. For the synthesis of Cu-poor CuPd tetrapods (CuPd TNP-2), we used the same procedure as described for CuPd TNP-1, changing only the amounts of precursors: 11.2 mg of Na_2_PdCl_4_ and 3.1 mg of CuCl.

### Instrumentation

TEM images were taken using a Hitachi H-7650 transmission electron microscope at an acceleration voltage of 100 kV. Energy dispersive X-ray (EDX) analysis was collected on a scanning electron microscope (SEM, JSM-6700F) using silicon wafer. X-ray diffraction (XRD) characterization was performed using a Philips X’Pert Pro X-ray diffractometer with a monochromatized Cu Kα radiation source and a wavelength of 0.1542 nm. The atomic ratio of Cu and Pd was investigated by inductively coupled plasma atomic emission spectrometry (ICP-AES, Atomscan Advantage, Thermo Jarrell Ash, USA). The MSOT equipment (inVision 128) was purchased from iThera Medical (Munich, Germany), and includes a total of 128 ultrasound transducer elements, each at 5 MHz, arranged in an array of 270 degrees. Illumination of the imaging plane is achieved with an OPO pumped Nd:YAG laser, which is tunable in 1 nm steps between 680 nm and 980 nm.

### Cell culture

Cell culture reagents were purchased from Gibco (Carlsbad, CA). All cells used in this research were cultured in DMEM (Invitrogen, 10% FBS, 100 µg mL^−1^ streptomycin, and 100 µg mL^−1^ penicillin) at 37 °C under 5 % CO_2_.

### EGFP-LC3 dot formation assay

EGFP-LC3/HeLa cells were observed under fluorescence microscopy after treatment, EGFP-LC3 dot formation was quantified by counting 300 cells and expressed as the ratio between the number of cells with at least five EGFP-LC3 dots and the number of cells with green fluorescence (essentially 100% for our cells stably expressing EGFP-LC3). The assays were independently performed by two of the authors in a blind manner.

### ROS detection

After treatments, cells were incubated for 20 min at 37 °C with 10 μM DCFH-DA in DMEM without FBS and antibiotic. After being washed three times in sterile PBS, cells were visualized byfluorescence microscope. And the mean fluorescence intensity was determined using aflow cytometer (BD Bioscience), and the data were analyzed using FlowJo software.

### Evaluation of lysosomal acidity

HeLa cells were collected after treating with CuPd TNP-2 for 24 h and washed twice in PBS. The cells were incubated in pre-warmed medium containing 1 uM LysoSensor Green DND-189 (Invitrogen, L-7535) dye for 30 min under growth conditions. The cells were resuspended in PBS after washing and analyzed by flow cytometry (FACS). FL1 (green) fluorescence was collected on a population of 10,000 cells.

### Cell viability assay

MTT was used to assess the cell viability. HeLa and 4T1 cells were grown in 96-well plates at a density of approximately 10,000 cells per well. After different treatments, MTT (thiazoyl blue tetrazolium bromide; T0793–500MG, Bio Basic) was added to the growing cultures at a final concentration of 0.5 mg mL^−1^ and incubated for 4 h at 37 C. Then the absorbance at 570 nm was measured with a spectrophotometer (Elx800, BioTek,Winooski, VT, USA).

### Photothermal killing of cancer cells

HeLa cells and 4T1 cells were seeded in 96-well plate with a density of 10,000 cells per well. Different concentration CuPd TNPs were added in wells and incubated with HeLa cells and 4T1 cells for 24 h. Then washed with cold PBS for twice and irradiated with 808 nm laser (1.2 W cm^2^^-1) for 3 min. The cell viabilities were determined by a standard MTT assay.

### Western blotting

The treated cells were harvested and lysed with the lysis buffer (1% Nonidet *P*-40 (Beyotime, ST366), 1% sodium deoxycholate (Pierce, #89905), 25 mM Tris-HCl, 150 mM NaCl, pH 7.6) supplemented with a protease inhibitor cocktail (Sigma, P8340) on ice. An equal volume of 2 × SDS sample buffer was added, and boiled for 10 min. Proteins were separated by electrophoresis on a SDS-polyacrylamide gel and transferred to PVDF membrane (Millipore, IPVH00010). After blocking with 5% nonfat dry milk for 1 h, the PVDF membrane was incubated for 2 h with a primary antibody at room temperature, extensively washed, incubated with horseradish peroxidase-conjugated secondary antibody (1:10,000 dilution) for 1 h, and finally visualized with an enhanced chemiluminescence (ECL) kit. Uncropped scans can be found in Supplementary Fig 23.

### Apoptosis assay

Apoptosis detection was performed with the Annexin-V-FLUOS Staining Kit (Roche, Cat.No.11858777001). Briefly, 20 μL Annexin-V-Fluos labeling reagent in 1 mL Incubation buffer and add 20 μL Propidium iodide solution. Cells were collected and washed with PBS and then resuspended in 100 μL of Annexin-V-FLUOS labeling solution. Incubate 10 min at 25 °C and then analyze by flow cytometer. Approximately 3 × 10^4^cells were analyzed in each of the samples.

### Tumor cell isolation from patients

Human breast infiltrating ductal carcinoma specimens (ER^-^PR^-^Her2^+^, grade III) used in this study were obtained from Nanfang Hospital (Guangzhou, Guangdong, China), while the patient had already received three rounds of neoadjuvant chemotherapy without any significant improvement. Therapeutic details were as follows: Gemcitabine d1/d8–1.6 g/1.6 g plus Xeloda (Capecitabine) d1/d14–1.5 g, 2/day, Vinorelbine Bitartrate Injection (NAVELBINE) d1/d8–40mg/40 mg plus Caelyx (doxorubicin HCl) 60 mg, Paclitaxel Liposome for Injection 270 mg plus Carboplatin 0.45 g plus Herceptin (Trastuzumab Injection) 600 mg. Human gastric fundus adenocarcinoma specimens were obtained from Sun Yat-sen University Cancer Center (Guangzhou, Guangdong, China). The experiments using patient-derived tumors have complied with all relevant ethical regulations and were performed according to the approved guidelines established by the Institutional Human Research Subjects Protection Committee of the Ethics Committee of the South China University of Technology, with the informed consent from the patients. The tumors obtained after surgery were immersed in a sterile 50 mL tube containing HBSS with 5% Antibiotic-Antimycotic, placed in ice and transport to lab within 4 h. The tumor tissue were washed with HBSS with antibiotic-antimycotic and cut into 1~2 mm^3^ fragments, then resuspended with 20 mL of tumor cell dissociation solution and incubated at 37 °C for 2 h with agitation. The suspension was passed through the 70 μm cell strainer, and the flow through was centrifuged at 500 xg for 5 min, with the cell pellet resuspended with medium for further culture.

### Phantom test

Cylindrical phantoms (provided with the MSOT equipment, operated following the instruction) were applied to test the linearity of the MSOT system as well as the relationship between concentration and MSOT signal. Different concentrations of CuPd TNP-2 were injected into the middle cavity of the phantom prepared by mixture of agar and intralipid, and the phantom was placed in the MSOT equipment. Images of the phantoms were taken at three different positions near the middle of the phantom, and data acquisition was performed using ten averages at 808 nm.

### MSOT imaging

Orthotopic 4T1 breast tumor-bearing mice are anaesthetized with 2% isofluorane throughout the experiments, and shaved with a hair clipper around the tumor. Then placed in a horizontal position in a holder surrounded by a thin polyethylene membrane to prevent direct contact with water and allow acoustic coupling between mouse and transducer array. The light fibers and ultrasonic transducer array are in a fixed position for all data acquisitions, whereas the mouse can be translated through the imaging plane using a linear stage. MSOT images were performed using ten averages at 680, 750, 808, 900 nm.

### Animal studies

BALB/c mice (female, 18–20 g, 5–6 weeks) and NOD/SCID mice (female, 18–20 g, 5–6 weeks) were purchased from the Beijing Vital River Laboratory Animal Technology Co., Ltd. (Beijing, China) and all animals received care in compliance with the guidelines outlined in the Guide for the Care and Use of Laboratory Animals. The 7-day treatment regimen was approved by the Animal Care and Use Committee of the University of Science & Technology of China (USTC) and conducted at the animal facility at the USTC, while the 15-day treatment regimen was approved by the Animal Care and Use Committee of South China University of Technology (SCUT) and conducted at the animal facility at the SCUT. About 100 μL of 4T1 (5 × 10^5^ cells) or MCF-7/MDR (1 × 10^7^ cells) in Matrigel (BD Biosciences) were orthotopic injected in the mammary fat pad of the BALB/c (for 4T1) and NOD/SCID (for MCF7/MDR) mice. The wild-type and *Atg5*-/- HeLa xenograft tumor model were generated by injecting 1 × 10^7^ wild-type or *Atg5*-/- HeLa cells in 100 μL PBS into the right flank of the NOD/SCID mice. For both the 7-day and 15-day regimen, PTT procedures were initiated when tumor volumes reached about 100 mm^3^, with mice receiving the indicated injection into the tail vein on day 1 and day 4 and getting PTT treatment (1.0 W cm^2^^-1 laser irradiation on the tumor site for 3 min) on day 2 and day 5. For ChemoPTT treatments, CQ or 3-MA were mixed with TNPs and co-injected into tail vein of mice, while Dox was given in separate injections. Thermographs and the tumor-site temperature changes were recorded during NIR irradiation by using an infrared thermal imaging camera, and the tumor volumes and body weights of mice were recorded every day. The tumor sizes were measured by a caliper and tumor volume (mm^3^) was calculated as V = lw^2^ 2^-1, in which l and w indicate the length and width of the tumor, respectively.

### Pharmacokinetics study

Female BALB/c mice were randomly divided into four groups (*n* = 5 per group). CuPd TNPs were intravenously injected at an equivalent dose of 1.5 mg CuPd TNPs per kg of mouse body weight. At predetermined time points (0, 0.5, 1, 2, 4, 8, 16, and 24 h), blood samples were collected from the retro-orbital plexus of the eye and then placed in heparinized tubes and centrifuged to obtain plasma. An equal volume of plasma was withdrawn, and the plasma samples were then nitrated. The platinum content in the plasma was detected by ICP-MS.

### Toxicity study

BALB/c mice were randomly divided into three groups (*n* = 5 per group), and treated intravenously with an equivalent dose of 1.5 mg CuPd TNPs per kg of mouse body weight following the procedures of therapeutic study in 4T1 tumor model. On the day after the last injection, the blood serum was collected and mouse alanine aminotransferase (ALT), aspartate transaminase (AST), blood urea nitrogen (BUN) and serum creatinine (SCR) were measured using quantitative enzyme-linked immunosorbent assay (ELISA) kits, following validation of each ELISA kit according to the manufacturer’s instructions. The major organs of the mice after treatment were collected and stained by haematoxylin & eosin (H&E) to evaluate the toxicities.

### TUNEL assay

Tumors that received various treatments were used for TUNEL assay (TUNEL BrightRed Apoptosis Detection Kit, A113–01, Vazyme). In all, 4 mm frozen sections were washed with PBS and then incubated with proteinase K for 3 min at room temperature. After incubation with equilibration buffer for 30 min, the TUNEL reaction mixture was added to rinsed slides and incubated in a humidified chamber for 60 min at 37 °C. After washing with PBS, the sections were stained for 10 min with DAPI, washed with PBS and visualized with a fluorescence microscope.

### Statistical analysis

All data were expressed as Mean ± s.e.m. and analyzed by two-tailed Student’s *t*-test. *p < 0.05, **p < 0.01 and ***p < 0.001 were considered statistically significant.

## Electronic supplementary material

Supplementary Information

## Data Availability

The data that support the findings of this study are available from the corresponding author upon request.
