# Supplementary material for: Harnessing copper-palladium alloy tetrapod nanoparticle-induced pro-survival autophagy for optimized photothermal therapy of drug-resistant cancer
Source: Nat Commun. 2018 Oct 12;9:4236. doi: 10.1038/s41467-018-06529-y (PMC6185906; doi:10.1038/s41467-018-06529-y)
Supplement: Supplementary file 1 — Supplementary Information [file 41467_2018_6529_MOESM1_ESM.pdf]

Supplementary Information

Harnessing Copper-Palladium Alloy Tetrapod Nanoparticle-induced Pro-survival Autophagy for Optimized Photothermal Therapy of Drug-resistant Cancer

Zhang *et al.*

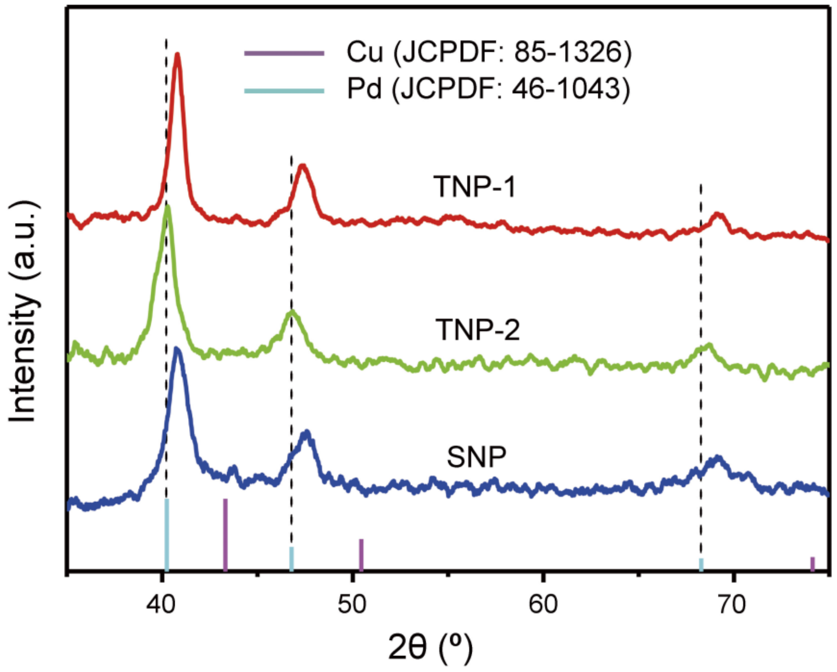

Supplementary Figure 1. XRD patterns of CuPd nanocrystals (CuPd TNP-1, TNP-2, SNPs).

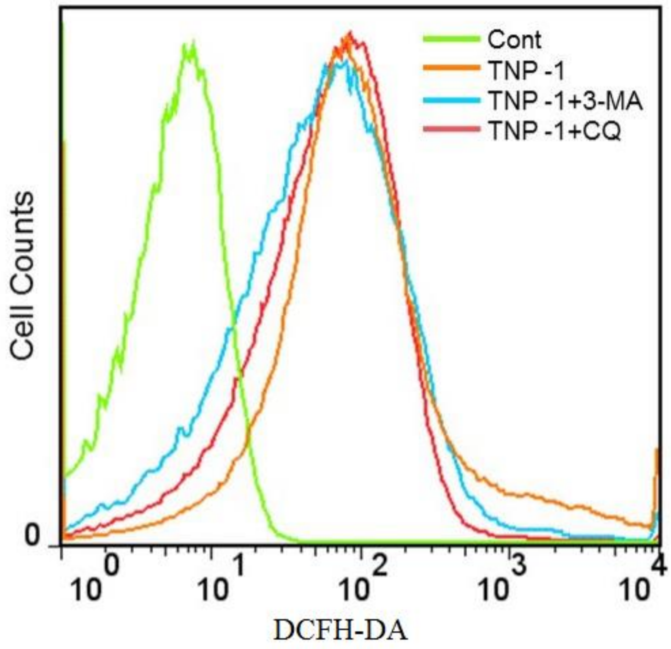

Supplementary Figure 2. FACS analysis of intracellular ROS after HeLa cells were treated with PBS (control), TNP-1 (10 μg/mL), or TNP-1 in the presence of 3-MA (2.5 mM) or CQ (25 mM) for 24 h.

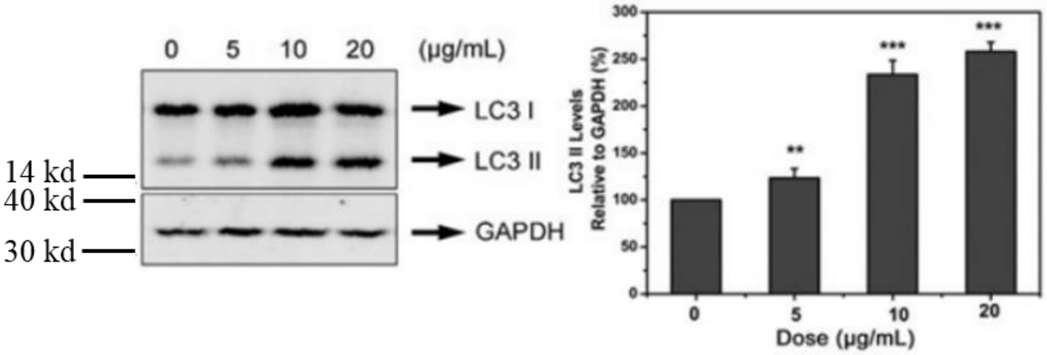

Supplementary Figure 3. HeLa cells were treated with the various doses of TNP-1 for 24 h and were subjected to Western blotting with anti-LC3 and GADPH antibodies. Right panels showed the quantified results. Mean ± s.e.m. n = 3. $^{**}p < 0.01$, $^{***}p < 0.001$.

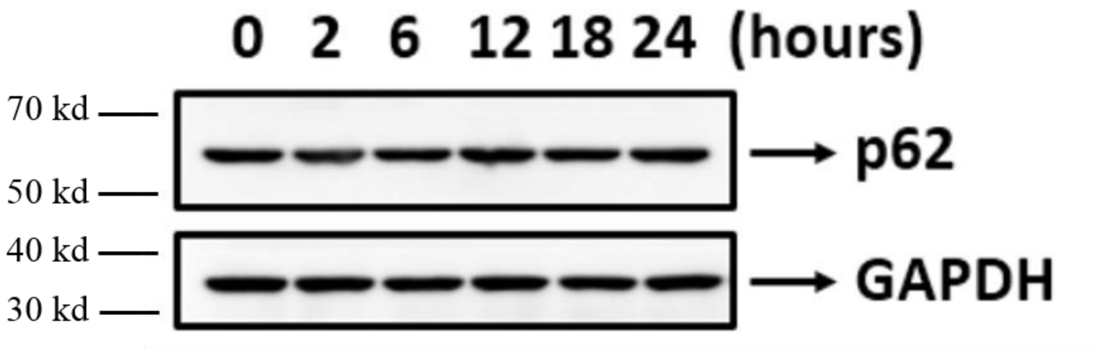

Supplementary Figure 4. Western blotting of p62 and GAPDH (served as loading control) in HeLa cells treated with 10 µg/mL CuPd TNP-2 for the various times.

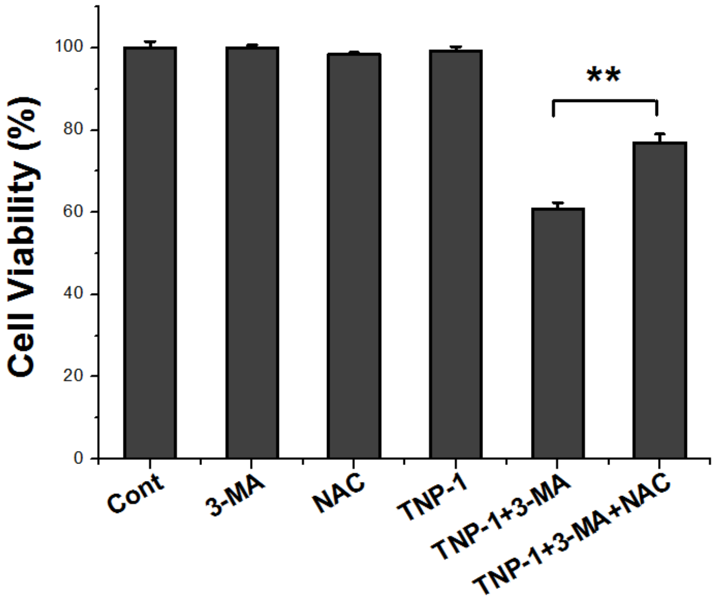

Supplementary Figure 5. Cell viability of HeLa cells after treatment for 24 h with the indicated combination. Dosing used: TNP-1: 10 µg/mL; 3-MA: 2.5 mM; NAC (N-acetyl cysteine): 25 mM. Mean ± s.e.m. n =5, **p < 0.01.

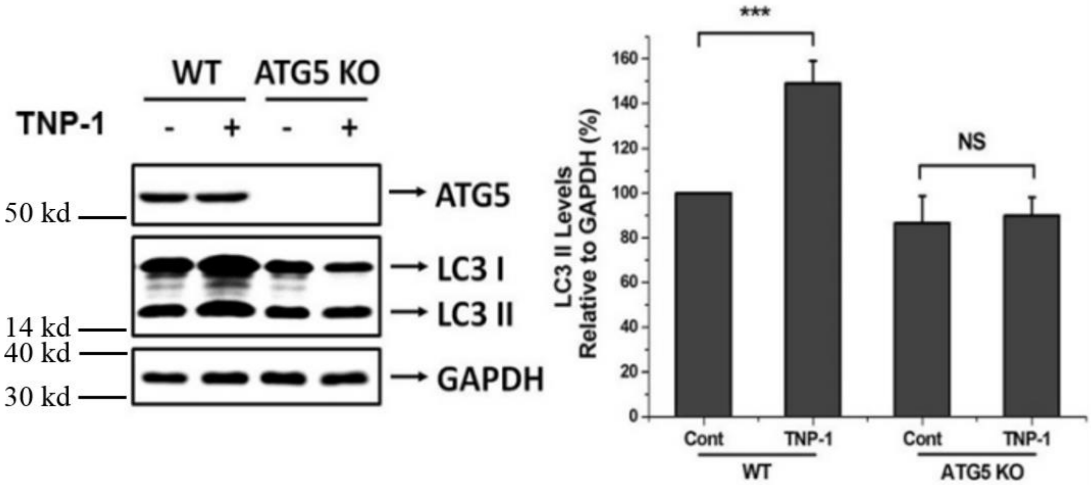

Supplementary Figure 6. Wild-type and ATG5 knockout (ATG5 KO) HeLa cells were treated with PBS (cont) or 10 μg/mL CuPd TNP-1 for 24 h and were subjected to Western blotting with antibodies against ATG5, LC3 or GADPH. Right panel showed the quantified densitometric analysis results. Mean ± s.e.m. n = 3. ***$p < 0.001$, NS: not significant.

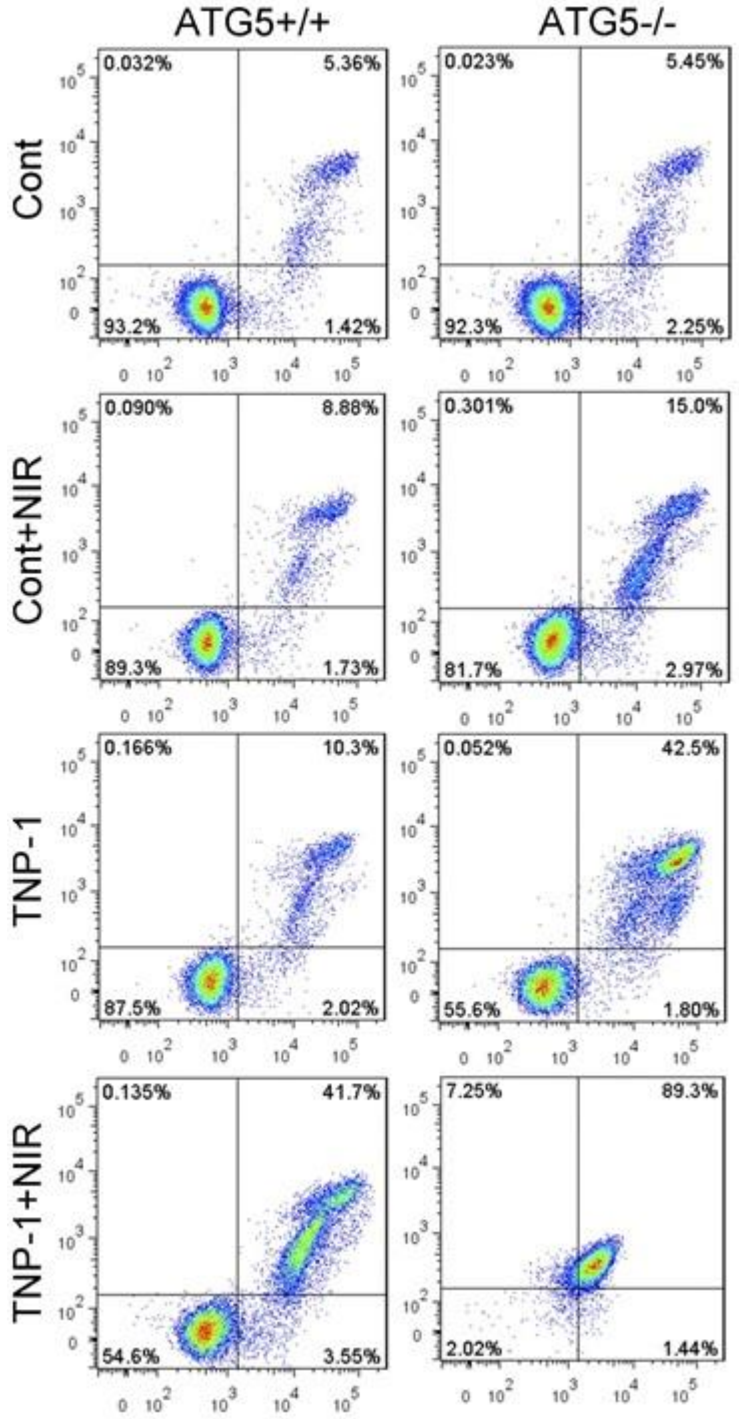

Supplementary Figure 7. Annexin-V/PI assay of wild-type and ATG5 knockout (ATG5-/-) HeLa cells treated with PBS (control) or 10 μg/mL CuPd TNP-1 for 24 h in the presence or absence of irradiation by 808-nm NIR laser at a power density of 1 W/cm^2 for 3 min. Mean ± s.e.m. n ≥ 5.

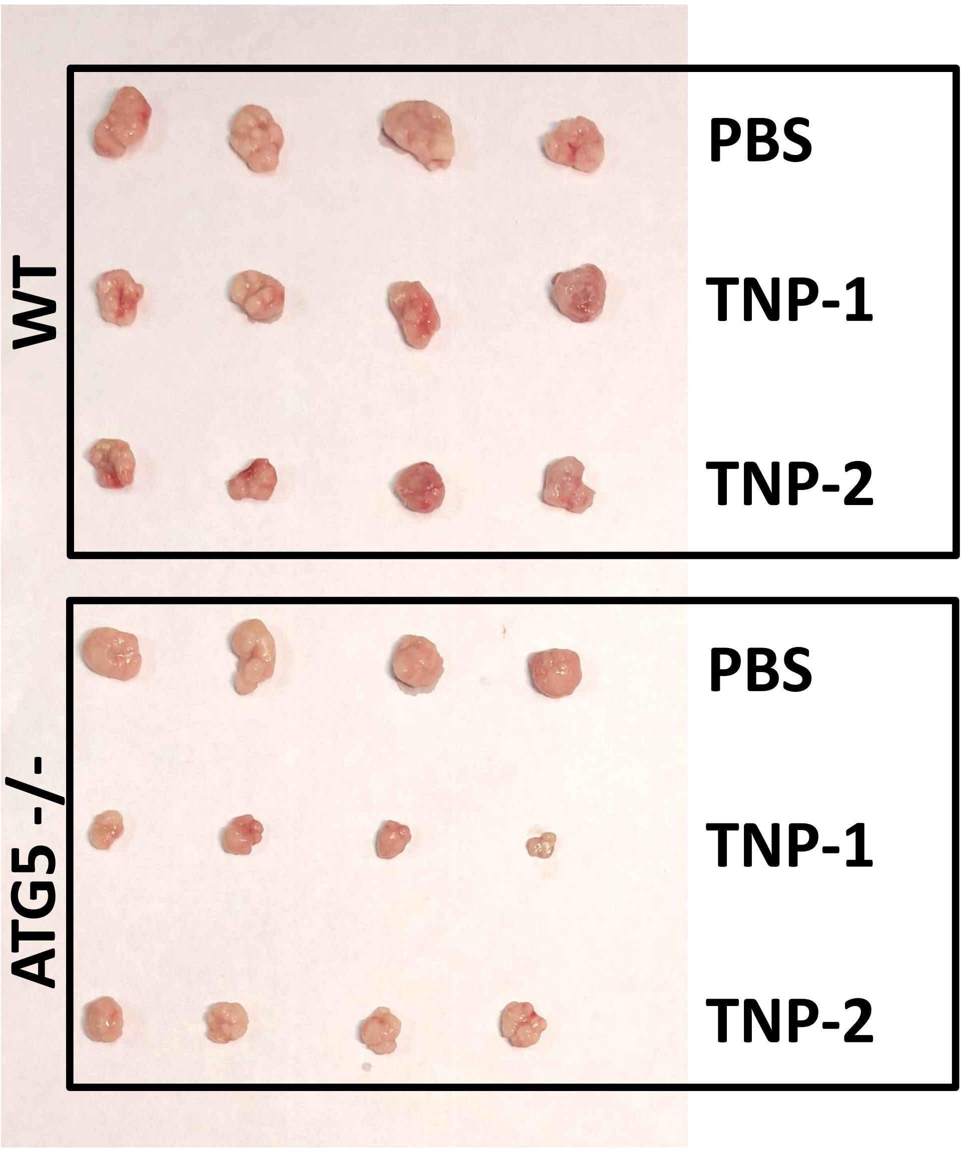

Supplementary Figure 8. Photographs of the excised tumors, after the 15-day PTT treatment regimen, in NOD/SCID mice harboring wild-type and ATG5 knock out HeLa tumors.

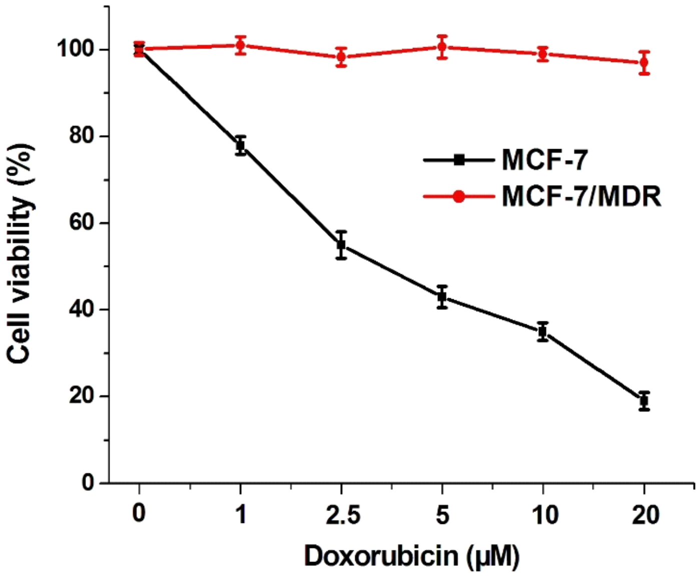

Supplementary Figure 9. Cell viability of regular and drug-resistant MCF-7 cells after treatment with different doses of doxorubicin (DOX) for 24 as determined by MTT assay. Mean ± s.e.m. n =5

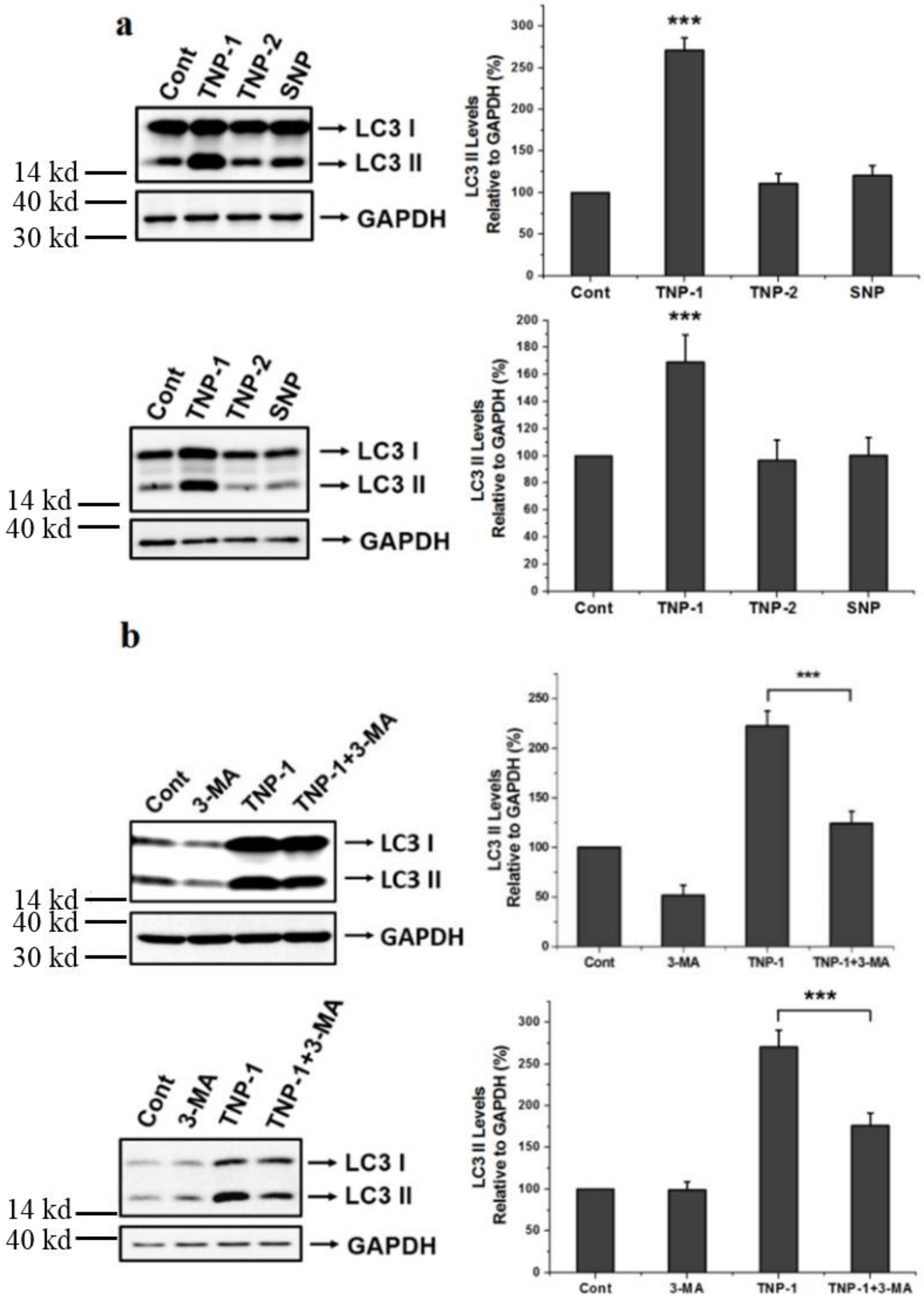
a
Cont
TNP-1
TNP-2
SNP
LC3 I
LC3 II
GAPDH
14 kd
40 kd
30 kd
LC3 II Levels
Relative to GAPDH (%)
***
b
3-MA
TNP-1+3-MA

Supplementary Figure 10. (a) Western blotting of LC3 and GAPDH (served as loading control) in 4T1 (top panel) and MCF-7/MDR (bottom panel) cells treated with 10 ug/mL CuPd TNP-1, CuPd TNP-2 or SNPs for 24 h. **(b)**Western blotting of LC3 and GAPDH (served as loading control) in 4T1 (top panel) and MCF-7/MDR (bottom panel) cells treated with 10 μg/mL CuPd TNP-1 for 24 h in the presence or absence of 3-MA.Endogenous LC3-II levels were detected by Western blotting with anti-LC3 antibodies and quantified by densitometric analysis relative to GAPDH. Mean ± s.e.m. n = 3. ***p < 0.001.

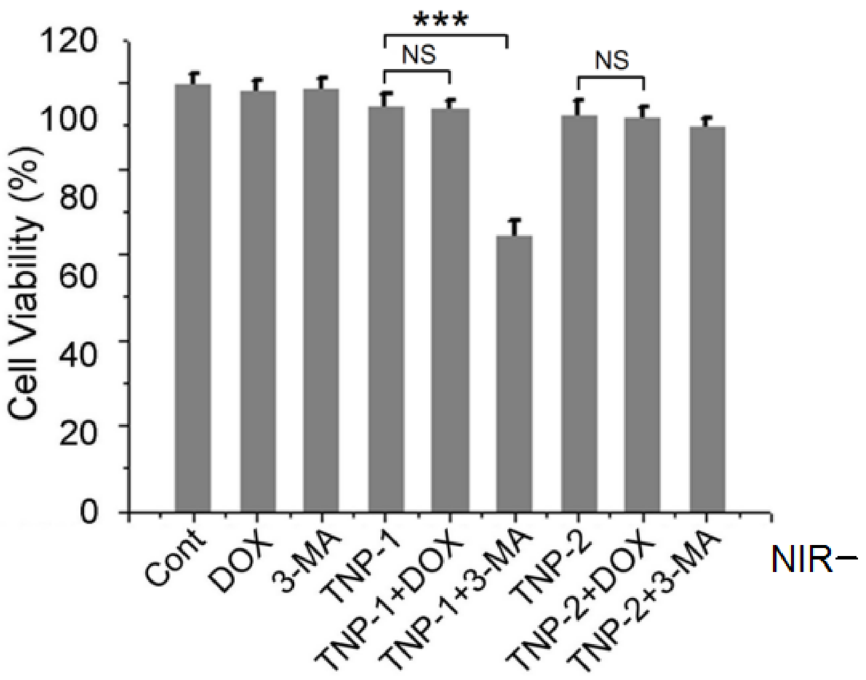

Supplementary Figure 11. Cell viability of MCF-7/MDR cells after treatment for 24 h with the indicated combination. Dosing used: TNP-1 or TNP-2: 10 μg/mL; 3-MA: 2.5 mM; Dox, 25 μM. Mean ± s.e.m. n =5, ***p < 0.001, NS: not significant.

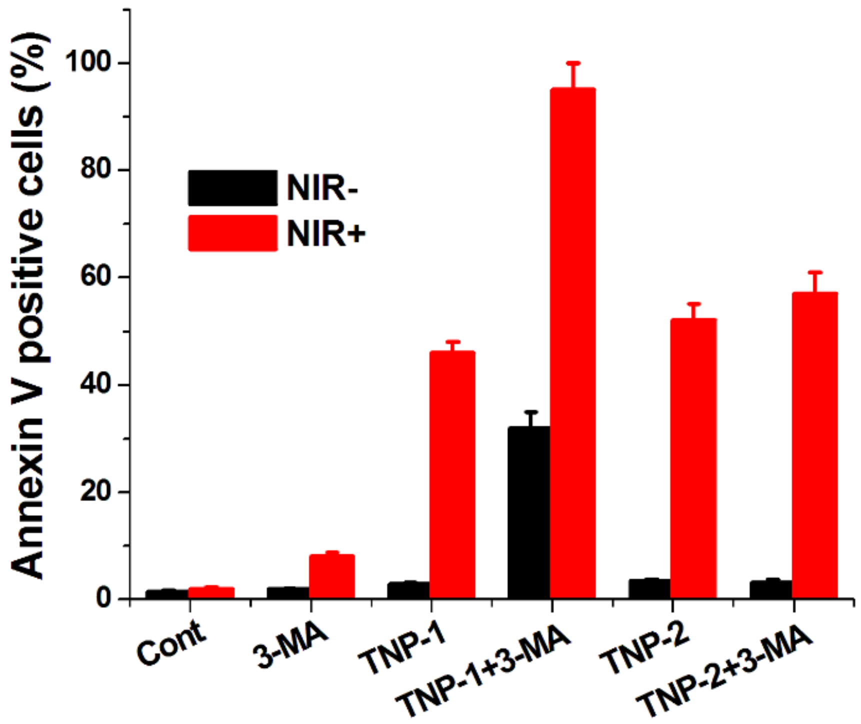

Supplementary Figure 12. Annexin-V/PI assay of 4T1 cells after the indicated treatment for 24 h. Dosing: 10 μg/mL CuPd TNP-1 or TNP-2; 2.5 mM 3-MA; Irradiation by 808 nm NIR laser at a power density of 1 W/cm^2 for 3 min. The ratio of Annexin-V positive cells was quantified by FACS analysis.

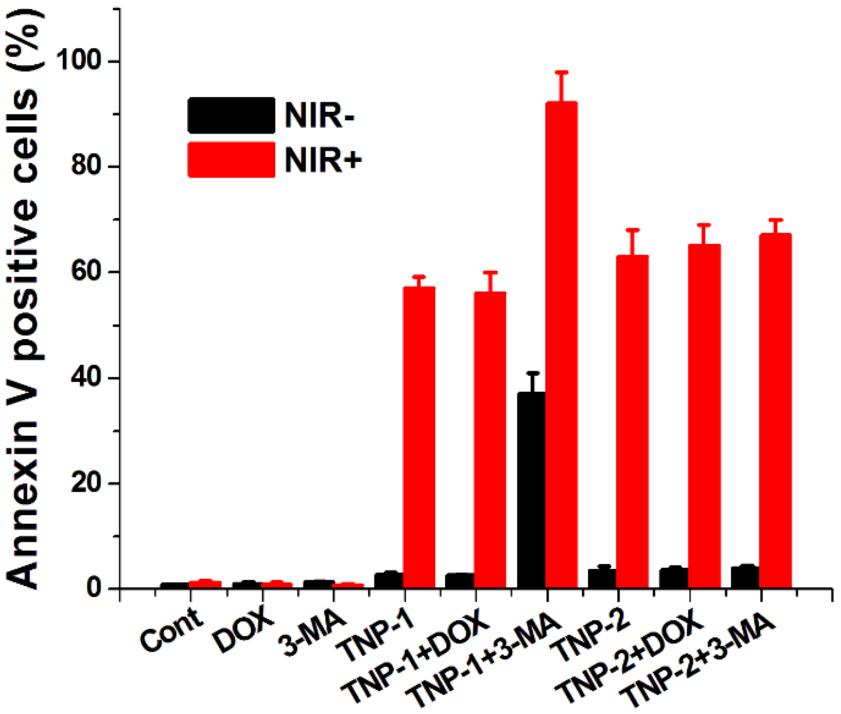

Supplementary Figure 13. Annexin-V/PI assay of MCF-7/MDR cells treated as indicated for 24 h. Dosing: 10 µg/mL CuPd TNPs; 2.5 mM 3-MA; 20 µM DOX; irradiation by 808-nm NIR laser at a power density of 1 W/cm^2 for 3 min. The ratio of Annexin-V positive cells was quantified by FACS analysis.

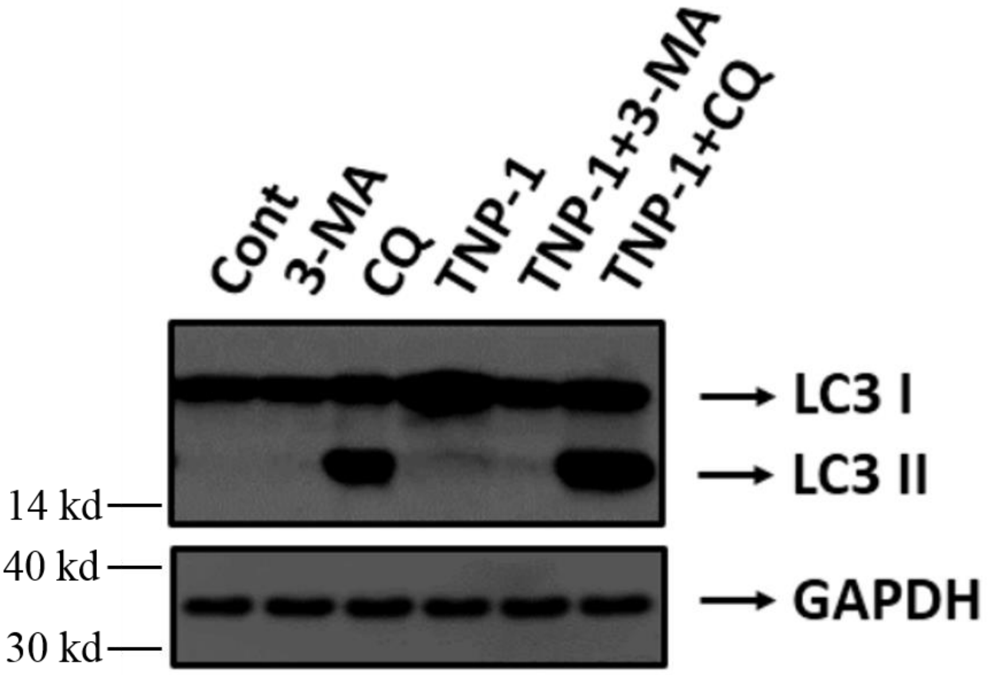

Supplementary Figure 14. Tumor cells derived from the gastric cancer patient were treated with 10 μg/mL CuPd TNP-1 for 24 h in the presence or absence of 3-MA and CQ and subject to Western blotting with antibodies against LC3 and GAPDH (served as loading control).

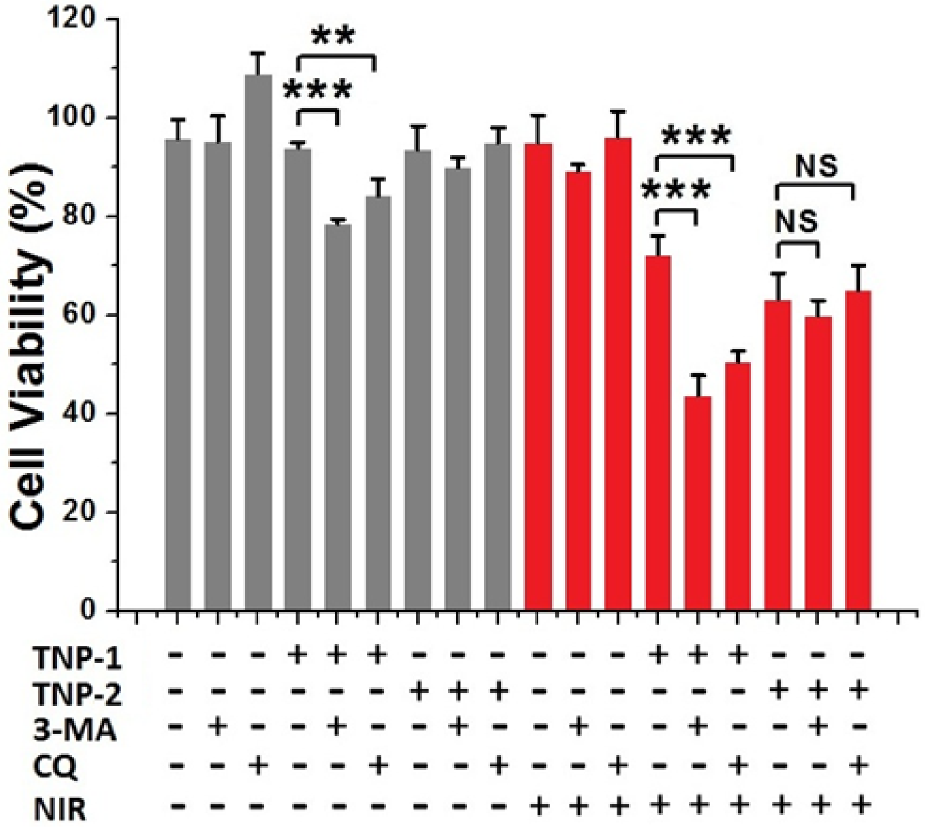

Supplementary Figure 15. Cell viability of tumor cells derived from the gastric cancer patient after the indicated treatment for 24 h. Dosing: 10 μg/mL CuPd TNP-1 or TNP-2; 2.5 mM 3-MA; 25 mM CQ; Irradiation by 808-nm NIR laser at a power density of 1 W/cm^2 for 3 min. Mean ± s.e.m. n = 5, **$p < 0.01$, ***$p < 0.001$, NS: not significant.

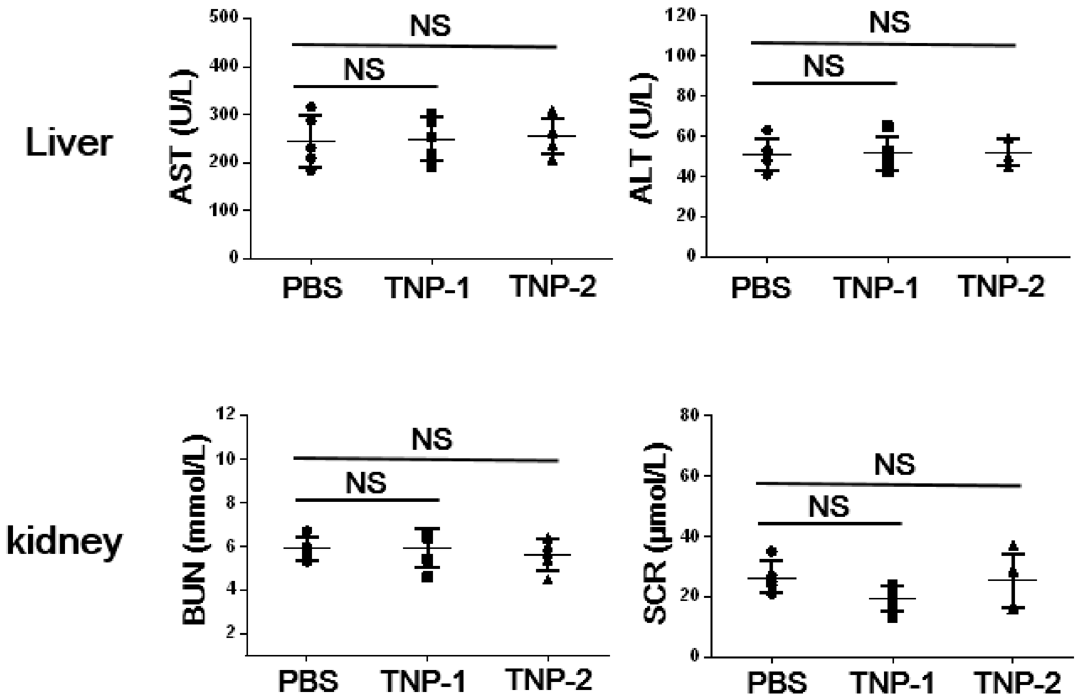

Supplementary Figure 16. Levels of liver ALT (Alanine aminotransferase) and AST (Aspartate aminotransferase) and kidney BUN (Blood urea nitrogen) and SCR (Serum creatinine) in Balb/c mice after treatment with PBS, 1.5 mg/kg TNP-1 or 1.5 mg/kg TNP-2 for 24 h. Mean ± s.e.m. n=5. NS: not significant.

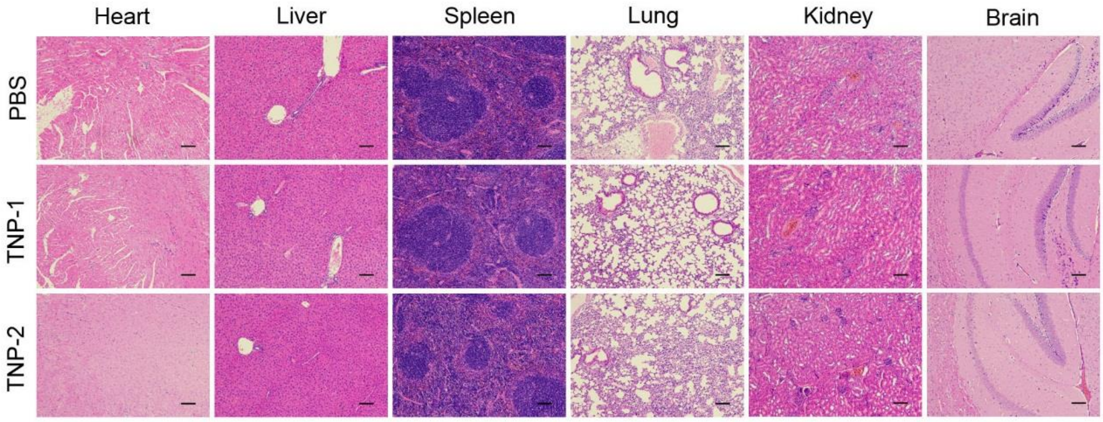

Supplementary Figure 17. Hematoxylin & eosin (H&E) staining of the major organs of Balb/c mice after treatment with PBS, 1.5 mg/kg TNP-1 or 1.5 mg/kg TNP-2 for 24 h. Scale bar: 100 μm.

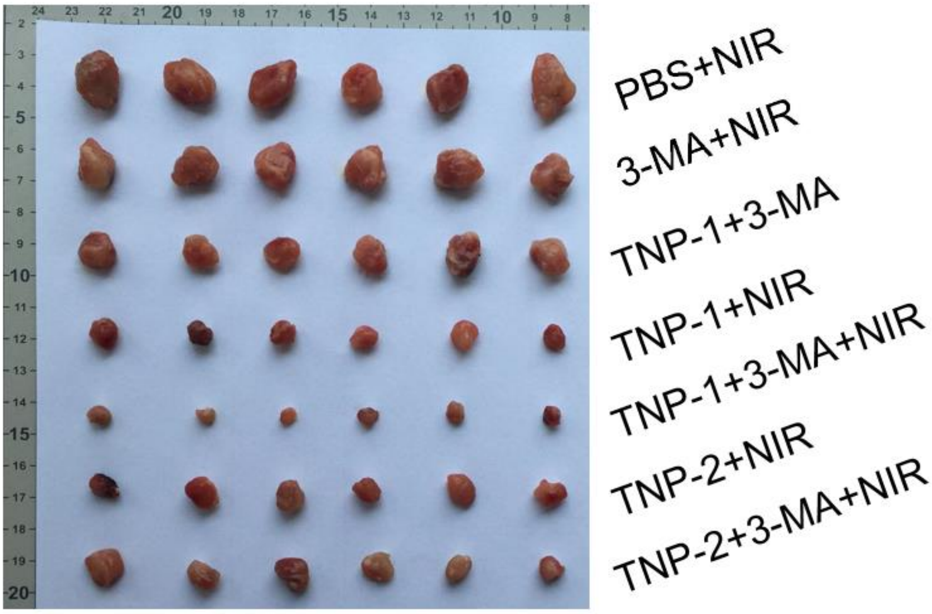

Supplementary Figure 18. Photographs of the excised tumors after the 7-day PTT treatment regimen in the orthotopic 4T1 breast cancer model. Dosing used: 100 μmol/kg 3-MA, 1.5 mg/kg TNP-1 or TNP-2.

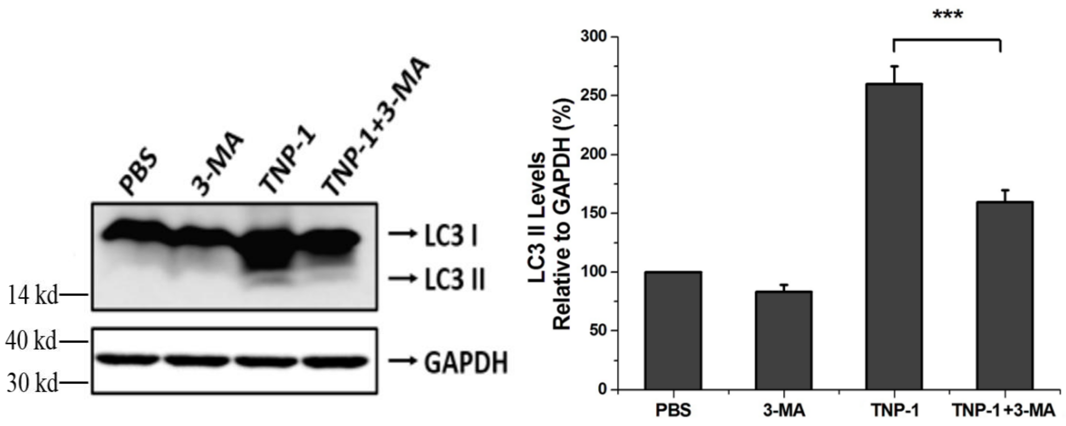

Supplementary Figure 19. Tumor tissues from the 4T1 tumor-bearing mice after the indicated PTT treatment were subjected to Western blotting with antibodies against LC3 and GADPH. Dosing used: 100 μmol/kg 3-MA and 1.5 mg/kg TNP-1. The right panel showed the quantified results. Mean ± s.e.m. n = 3. ***$p < 0.001$.

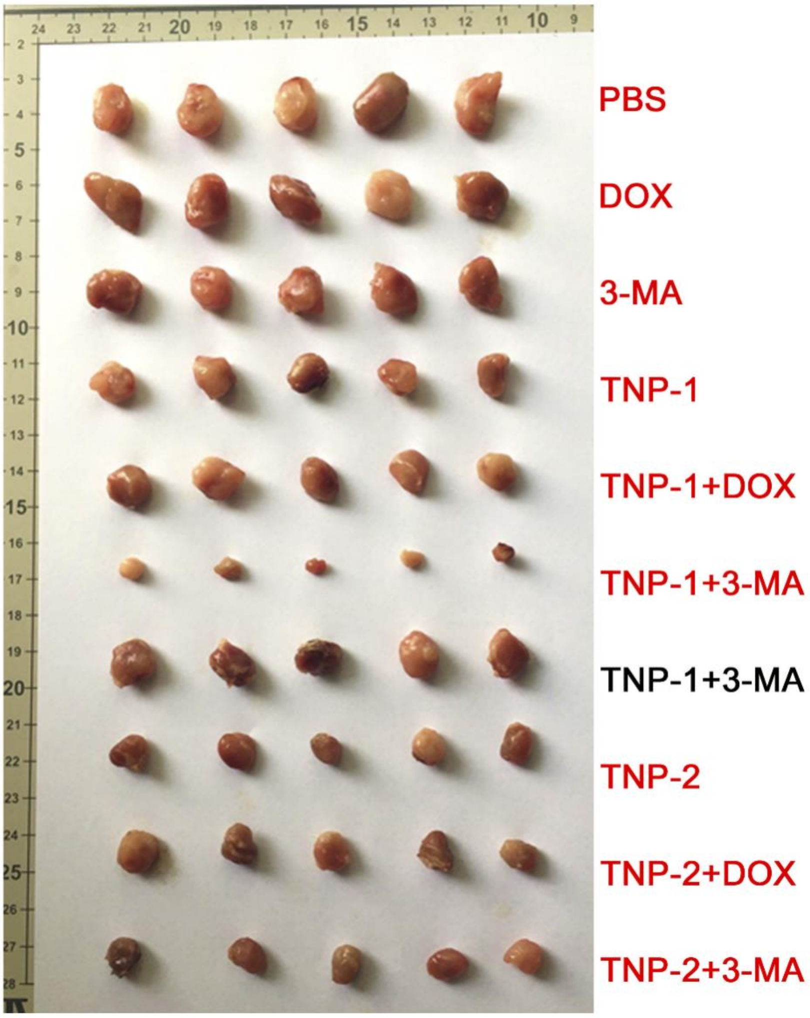

Supplementary Figure 20. Photographs of the excised tumors after the 7-day PTT treatment regimen in the orthotopic MCF7/MDR breast cancer model. Treatments marked in red indicated NIR irradiation, while one group marked in black (TNP-1 + 3-MA) did not receive NIR irradiation. Dosing used: 5 mg/kg DOX; 100 μmol/kg 3-MA, 1.5 mg/kg TNP-1 or TNP-2.

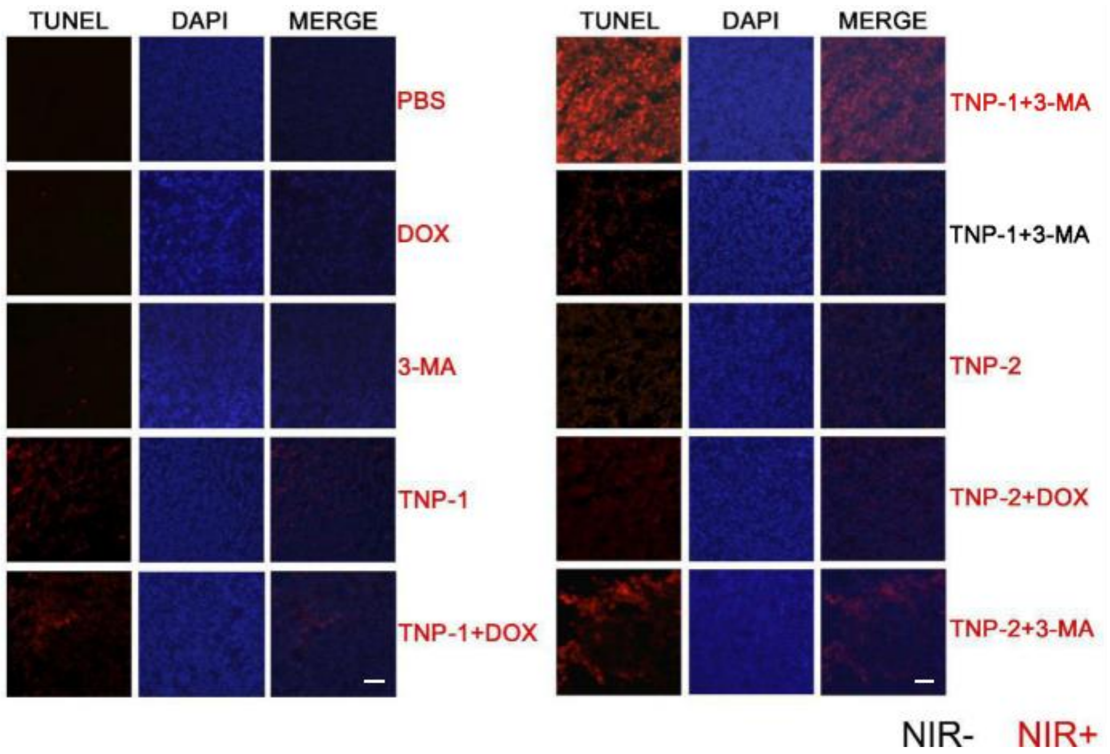

Supplementary Figure 21. TUNEL staining (red) of sections from tumors in Supplementary Figure 20 was performed to show apoptotic cells. Nucleus was stained with DAPI (blue). Scale bar, 50 μm.

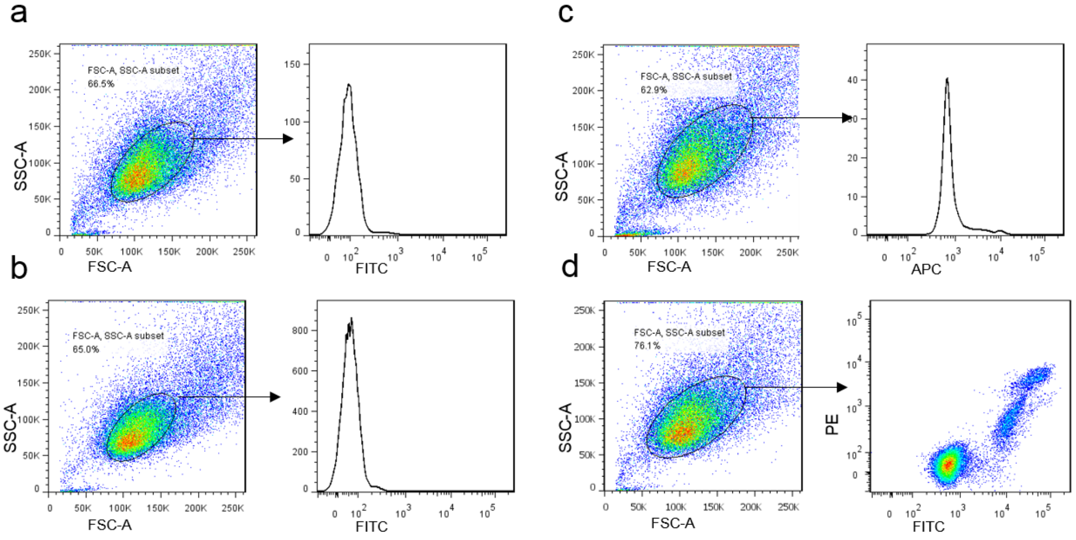

Supplementary Figure 22. Gating strategies for FACS. (a) Starting cells were gated by FSC/SSC gates. Representative histogram plots from Fig.3 a,b showing DCFH-DA staining for HeLa cells. (b) Starting cells were gated by FSC/SSC gates. Representative histogram plots from Fig. 3f showing LysoSensor Green DND-189 staining for HeLa cells. (c) Starting cells were gated by FSC/SSC gates. Representative histogram plots from Fig. 3g showing Magic Red Cathepsin-B staining for HeLa cells. (d) Starting cells were gated by FSC/SSC gates. Representative histogram plots from Fig. 4b showing Annexin -V- FLUOS and Annexin -V- Alexa 568 staining for HeLa cells.

Figure 2b

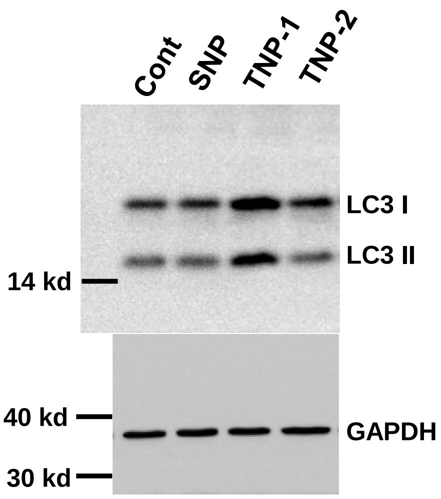

Figure 2d

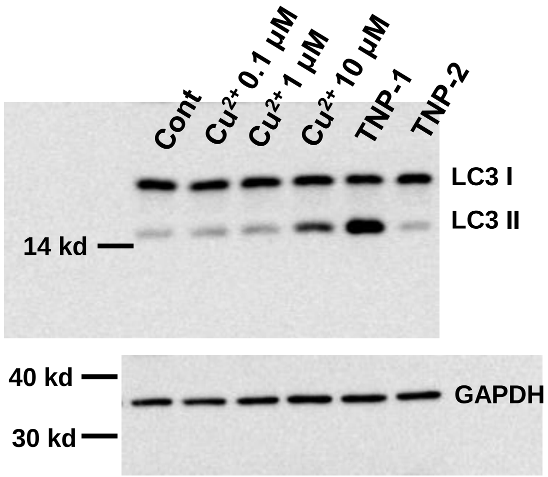

Figure 2e

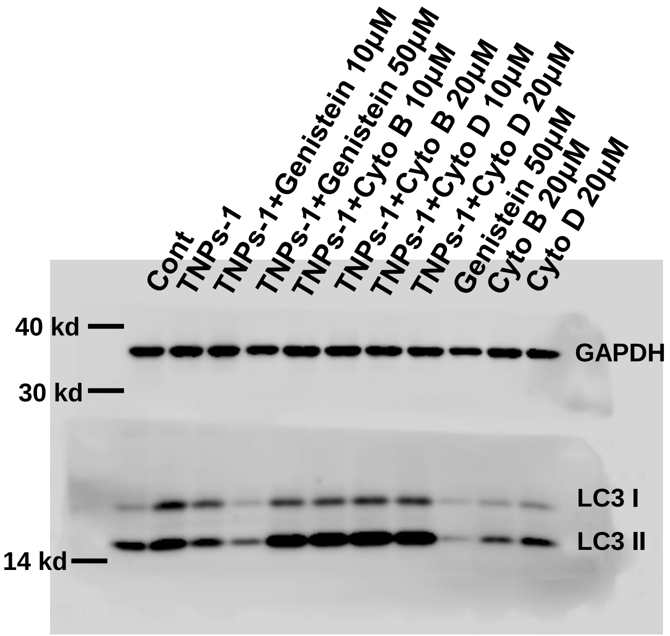

Figure 3c

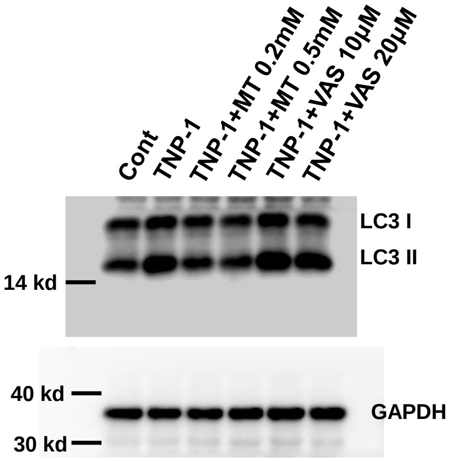

Figure 3d

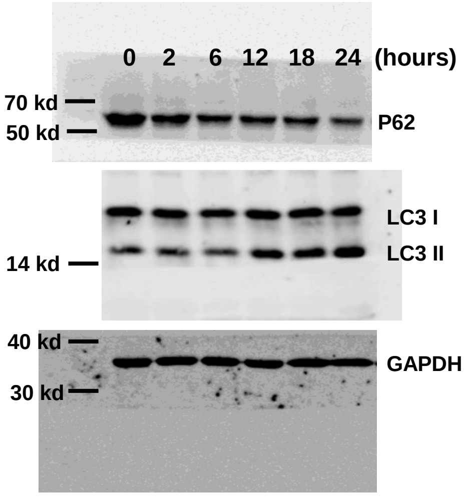

Figure 3e

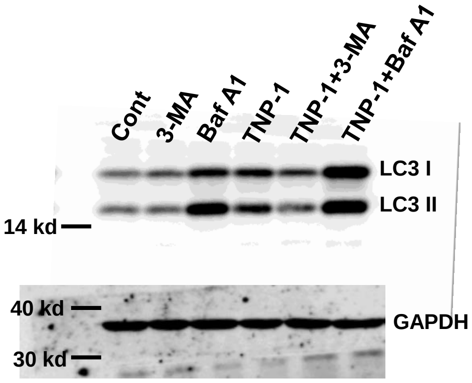

Figure 4g

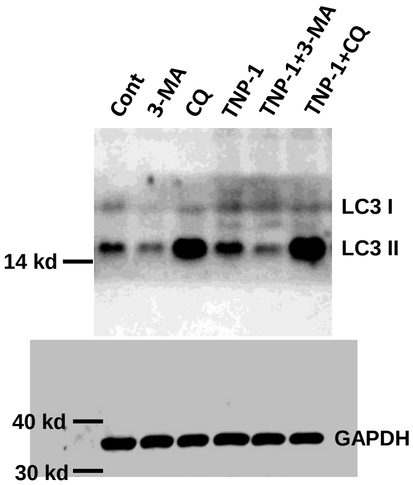

Supplementary Figure 23. Uncropped scans of Western blot with molecularweight markers. Related figure is indicated in red.

Supplementary Table 1.

Sample	Viable cells	Early apoptotic cells	Late apoptotic cells	Necrotic cells
Cont	97.7±0.2	0.7±0.1	1.4±0.6	0.2±0.1
3-MA	96.1±0.2	1.4±0.6	2.3±0.3	0.9±0.1
TNP-1	97.3±0.1	0.9±0.6	1.7±0.2	0.2±0.1
TNP-1+3-MA	67.0±0.5	1.7±0.3	28.7±0.4	2.6±0.4
TNP-2	96.4±0.2	0.9±0.1	2.1±0.5	0.5±0.4
NIR	97.4±0.2	0.9±0.1	1.6±0.4	0.1±0.1
3-MA+NIR	84.5±0.2	2.2±0.1	11.4±0.6	2.4±0.5
TNP-1+NIR	65.2±0.8	1.7±0.2	30.4±0.3	2.9±0.1
TNP-1+3-MA+NIR	1.0±0.4	0.8±0.5	91.6±0.3	6.7±0.3
TNP-2+NIR	43.5±0.5	9.3±0.2	39.9±0.2	7.4±0.2

Supplementary Table 1. Summarized data for the Annexin-V/PI assay of HeLa cells after treatment for 24 hour with the indicated combination, showing the relative percentage of cells. TNP-1 or TNP-2: 10 μg mL^-1; 3-MA: 2.5 mM; NIR irradiation: 1 W cm2^-1, 3 min.

Supplementary Table 2.

Cells	Sample	Viable cells	Early apoptotic cells	Late apoptotic cells	Necrotic cells
ATG5+/+	Cont	93.2±0.3	1.4±0.6	5.4±0.2	0.1±0.1
	Cont+NIR	89.3±0.4	1.7±0.2	8.9±0.6	0.1±0.1
	TNP-1	87.5±0.2	2.0±0.4	10.3±0.5	0.2±0.2
	TNP-1+NIR	54.6±0.7	3.5±0.1	41.7±0.4	0.1±0.2
ATG5-/-	Cont	92.3±0.2	2.3±0.3	5.5±0.4	0.1±0.0
	Cont+NIR	81.7±0.5	3.0±0.2	15.0±0.6	0.3±0.1
	TNP-1	55.6±0.4	1.8±0.1	42.5±0.5	0.1±0.3
	TNP-1+NIR	2.0±0.4	1.4±0.3	89.3±0.6	7.3±0.4

Supplementary Table 2. Summarized data for the Annexin-V/PI assay of wild-type and *ATG5* knockout (ATG5-/-) HeLa cells after treatment for 24 h with the indicated combination, showing the relative percentage of cells. TNP-1 or TNP-2: 10 μg mL^-1; NIR irradiation: 1 W cm2^-1, 3 min. Mean ± s.e.m. n = 5.

Supplementary Table 3. Pharmacokinetic parameters of TNP-1 and TNP-2 after intravenous administration (Mean ± s.e.m. n = 5).

	T1/2 (h)	AUC(0-24 h)[a] (μg L^-1*h)	Cmax[b] (μg L^-1)	MRT(0-24 h)[c] (h)
TNP-1/Pd	73.5±7.0	778.5±50.4	38.2±2.6	12.2±0.3
TNP-2/Pd	68.0±4.2	761.8±61.0	43.0±4.6	12.4±0.3

[a]Area under the curve (AUC).

[b]The maximum drug concentration (Cmax).

[c]Mean residence time (MRT). All the pharmacokinetic parameters were analyzed according to a non-compartment model by DAS 3.0.
